# Prediction models for immune checkpoint inhibitor-related cardiovascular toxicity: a systematic review

**DOI:** 10.3389/fimmu.2026.1903131

**Published:** 2026-08-19

**Authors:** Miaomiao Zhang, Defa Zhang, Kai Jiang, Qian Wang

**Affiliations:** 1 Department of Health and Management, Taishan Vocational College of Nursing, Taian, China; 2 Ward 2, Gastroenterology Department, Shandong Cancer Hospital and Institute, Shandong First Medical University and Shandong Academy of Medical Sciences, Jinan, China; 3 Department of Emergency Critical Care Medicine, Yantai Yuhuangding Hospital, Yantai, China; 4 Department of Anesthesiology and Operating Room, Shandong Cancer Hospital and Institute, Shandong First Medical University and Shandong Academy of Medical Sciences, Jinan, China

**Keywords:** cardiovascular toxicity, immune checkpoint inhibitor, neoplasm, prediction models, systematic review

## Abstract

**Background:**

Immune checkpoint inhibitor (ICI)-related cardiovascular toxicity is associated with high mortality, and prediction models may facilitate its prevention and management. However, the predictive performance, quality, risk of bias, and applicability of existing models remain unclear.

**Objective:**

To systematically review prediction models for ICI-related cardiovascular toxicity.

**Methods:**

Nine databases were searched for studies on prediction models for ICI-related cardiovascular toxicity from inception to July 12, 2026. Data were extracted in accordance with the Checklist for Critical Appraisal and Data Extraction for Systematic Reviews of Prediction Modelling Studies. The included models were assessed using the Quality, Risk of Bias, and Applicability Assessment Tool for Prediction Models Using Regression or Artificial Intelligence Methods. This systematic review was registered on PROSPERO (CRD420261296378).

**Results:**

A total of 34, 541 articles were identified, and 23 articles met the inclusion criteria. These 23 articles described 82 prognostic models (71 for predicting the occurrence risk of ICI-related cardiovascular toxicity and 11 for predicting the prognosis of this toxicity) and two diagnostic models. Modeling methods comprised logistic or Cox regression and machine learning algorithms. Common predictors included demographic characteristics, medical history, treatment-related characteristics, and laboratory and imaging parameters. The area under the receiver operating characteristic curves or C statistic ranged from 0.699 to 0.967 for apparent performance, 0.595 to 0.942 for internal performance, and 0.549 to 0.949 for external performance. Calibration and clinical utility were assessed in 20 and 15 models using calibration curves/Hosmer–Lemeshow test and decision curve/clinical impact curve analysis, respectively, in apparent, internal, or external performance validation. Models were presented using techniques such as nomograms, scoring systems, and regression equations. All models were graded as low quality, high risk of bias, and only seven models were graded as high applicability.

**Conclusions:**

Existing prediction models have concerns regarding quality, risk of bias, and applicability, limiting their clinical practice. Currently, baseline and dynamic risk screening using relevant risk biomarkers is suggested to better manage ICI-related cardiovascular toxicity. Future research is warranted to validate and improve existing models or develop new prediction models with rigorous methods.

**Systematic review registration:**

https://www.crd.york.ac.uk/PROSPERO/, identifier CRD420261296378.

## 1 Introduction

Immune checkpoint inhibitors (ICIs), including programmed cell death protein 1, programmed death-ligand 1, and cytotoxic T lymphocyte-associated antigen-4 inhibitors, can effectively promote complete or partial tumor remission and prolong patients’ progression-free and overall survival (1–7). Nevertheless, ICIs may trigger immune-related adverse events (irAEs) (8–11). ICI-related cardiovascular toxicity has attracted growing clinical attention. ICI-related cardiovascular toxicity is a composite concept, including arrhythmias, myocarditis, acute heart failure, atherosclerosis, acute coronary syndrome, pericarditis, and venous thromboembolism (12–14). Despite its relatively low incidence, this toxicity may result in temporary or permanent ICI discontinuation, trigger major adverse cardiovascular events, and even cause death (14–16). Notably, although the mortality of ICI-associated myocarditis has declined, it still exceeds 20%, rendering it one of the most important irAEs (14).

The Heart Failure Association and Cardio-Oncology Council of the European Society of Cardiology (ESC) recommend identifying patients at high risk of immunotherapy-related cardiovascular toxicity and delivering stratified cardiac screening and protection strategies (16). Relevant guidelines also advocate pre-treatment assessment of patients’ susceptibility to irAEs (8). Moreover, irAEs cause uncertainty to some patients and impair their quality of life (17, 18). All these evidences highlight the urgent need for risk prediction of ICI-related cardiovascular toxicity.

Treatment-specific prediction models provide personalized estimates of therapeutic benefits, risks, and prognoses, easing patients’ concerns over treatment outcomes. Such prediction tools facilitate doctor–patient communication and shared decision-making while enabling early identification and targeted management of high-risk populations (19–21). In recent years, an increasing number of prediction models for ICI-related cardiovascular toxicity have been developed (15, 19, 22–42), supporting clinical risk stratification and individualized monitoring. Nevertheless, insufficient evidence on predictive performance, quality, risk of bias, and clinical applicability substantially limit their clinical application. Systematic reviews are conducive to addressing these issues. Existing systematic reviews on prediction models for cancer treatment-related cardiovascular toxicity mainly focus on chemotherapy, radiotherapy, surgery, and targeted therapy (43–46), but no comprehensive systematic review has been performed on models for ICI-related cardiovascular toxicity.

This systematic review aims to evaluate studies on the development and validation of prediction models for ICI-related cardiovascular toxicity, encompassing overall ICI-related cardiovascular toxicity and individual subtypes such as ICI-related myocarditis and ICI-related thromboembolism. Moreover, it covers prognostic models for assessing the occurrence risk and prognosis of ICI-related cardiovascular toxicity, as well as diagnostic models for grading the severity of ICI-related cardiovascular toxicity.

## 2 Method

This systematic review was reported in line with the transparent reporting of multivariable prediction models for individual prognosis or diagnosis: checklist for systematic reviews and meta-analyses (TRIPOD-SRMA) (47) and registered on PROSPERO (CRD420261296378).

### 2.1 Search strategy

First, the PICOTS system (48) was adopted to develop a clear search strategy and inclusion and exclusion criteria. Population: cancer patients treated with ICIs; Index models: prognostic prediction models for predicting the occurrence risk and prognosis of ICI-related cardiovascular toxicity, as well as diagnostic prediction models for grading the severity of ICI-related cardiovascular toxicity; Comparator models: not applicable; Outcomes: ICI-related cardiovascular toxicity and its prognosis; Timing and Setting: no restrictions.

Secondly, China National Knowledge Infrastructure, China Science and Technology Journal Database, Wanfang Database, Chinese Biomedical Literature Database, Chinese Medical Association Full-Text Journal Database, PubMed, Web of Science, Embase, and Cochrane Library were searched from inception to July 12, 2026. The core search terms focused on immune checkpoint inhibitors, ICI-related cardiovascular toxicity, and prediction models (see Supplementary Document for Literature Search Strategy). Furthermore, we screened the reference lists of included studies and relevant reviews to identify eligible literature.

### 2.2 Inclusion and exclusion criteria

Inclusion criteria included the following: Study design: cohort, case-control, cross-sectional, and intervention studies. Population: adult cancer patients receiving ICI treatment. Type of prediction model: prognostic prediction models for predicting the occurrence risk and prognosis of ICI-related cardiovascular toxicity and diagnostic prediction models for grading the severity of ICI-related cardiovascular toxicity. Exclusion criteria included the following: research protocols, reviews, conference abstracts, and commentaries; studies that focus only on potential predictors without forming prediction models, those that lack detailed model development processes or have overly simplistic development processes in model development studies, and those that have been repeatedly published.

### 2.3 Study selection

First, duplicates in retrieved records were eliminated via NoteExpress software and manual screening. Two evidence-based medicine-trained researchers independently screened titles and abstracts to exclude ineligible studies. Then, final eligibility was confirmed by full-text retrieval and detailed reading, and any disagreements were resolved by consulting a third researcher.

### 2.4 Data extraction

In accordance with the Checklist for Critical Appraisal and Data Extraction for Systematic Reviews of Prediction Modelling Studies (CHARMS checklist) (49), two researchers independently extracted basic study and model information, including author, publication year, country, study design, data source, research setting, patient characteristics, outcomes and their definitions or diagnostic criteria, prediction time, candidate predictors, variable screening methods, modeling methods, continuous variable processing methods, missing data details, model validation strategies, predictive performance metrics (such as calibration, discrimination, overall model fit, and clinical utility.etc), final predictors, and model presentation formats. Disagreements were addressed by consulting a third researcher.

### 2.5 Quality, risk of bias, and applicability assessment

Two researchers evaluated the included prediction models using the Quality, Risk of Bias, and Applicability Assessment Tool for Prediction Models Using Regression or Artificial Intelligence Methods (PROBAST+AI) (48). PROBAST+AI comprises quality, risk of bias, and applicability assessment parts during the model development and validation stage. The quality and risk of bias assessment parts include four domains: participants and data sources, predictors, outcome, and analysis. The first three domains include model applicability assessment questions. First, reviewers evaluated the quality, risk of bias, and applicability of each domain and then conducted a comprehensive assessment of the overall model, with disagreements resolved by consultation with a third researcher.

### 2.6 Data synthesis

Considering the heterogeneity of the prediction models included in this study, the results were summarized using a narrative synthesis method. We summarized the study and model information and assessed quality, risk of bias, and applicability of included models. The assessment results concerning quality, risk of bias and applicability were presented as heatmaps using R x64 4.1.0 software.

## 3 Result

### 3.1 Study selection and characteristics

**Figure 1:**
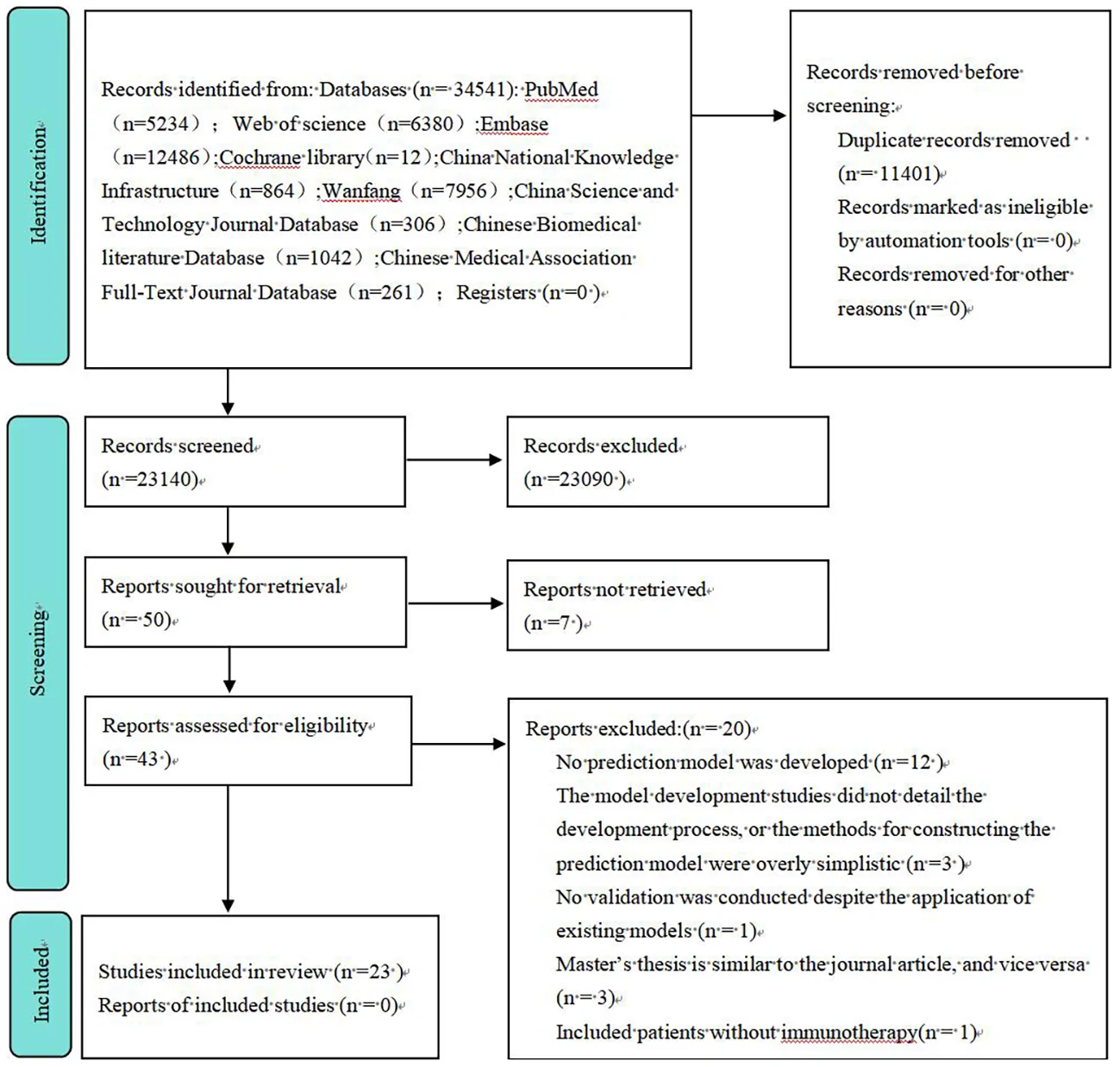
Flow diagram of the literature screening process.

A total of 34, 541 articles were retrieved, with 23 ultimately included (see **Figure 1** for study selection). These 23 articles, published between 2021 and 2026, included seven in Chinese (22–28) and 16 in English (15, 19, 29–42). Among them, 19 were from China (19, 22–28, 30–32, 34–38, 40–42), one from the USA, France, etc. (15), one from Japan and the USA (39), and two from the USA (29, 33). Twenty-two were retrospective studies (15, 19, 22, 24–42), and one was a prospective observational study (23). Additionally, 22 articles covered model development and validation (15, 19, 22–40, 42), and one focused only on validating existing models (41). All models were applied in healthcare settings (see **Table 1** for study characteristics).

**Table 1:** Study characteristics.

No	Author (year)	Country	Objective	Model number	Study design	Data sources and participants
1	Sha qi Rao 2026 (26)	China	To investigate the risk factors for myocarditis in tumor patients treated with ICIs and to construct a clinical prediction model.	Model 1	Single center retrospective study	Between October 2021 and October 2024; First Affiliated Hospital of Nanchang University; Tumor patients who received ICIs therapy.
2	Tao Luan 2026 (37)	China	To develop and externally validate a clinically accessible risk-prediction model based on routine baseline cardiac assessments to enable early identification and risk stratification of patients at high risk of severe ICI-associated myocarditis around ICI initiation.	Model 1	Multicentre retrospective study	Between January 2018 and June 2024; The First Affiliated Hospital of Guangzhou Medical University, Zhejiang Cancer Hospital, and The Affiliated Hospital of Kunming University of Science and Technology; patients with advanced lung cancer who received ICIs.
3	Zhuoling Zheng 2026 (42)	China	To identify risk factors and develop models for predicting both the occurrence and severity of ICI-associated myocarditis.	Model 1-2	Single center retrospective study	Between January 2020 and December 2024; The Sixth Affiliated Hospital, Sun Yat-sen University; Patients with malignant tumors who received ICI therapy.
4	John R. Power 2026 (15)	USA, France, etc	To determine predictors and construct a risk score associated with negative outcomes in patients admitted for ICI myocarditis.	Model 1	Multicentre retrospective study	Training set:Between 2014 and 2023; A retrospective registry spanning 127 institutions across 17 countries; Patients with ICI myocarditis. External validation set:(1)Between April 2023 and April 2024; Sorbonne University (SU, Paris, France); Patients with ICI myocarditis. (2)Between January 2015 and June 2022; Mass General Brigham (MGB, Boston, USA); Patients with ICI myocarditis.
5	Reina Yamamoto 2026 (39)	Japan and USA	To develop machine learning-based models to predict the onset of ICI-induced myocarditis within 3 months of starting ICI therapy, using a large health insurance database.	Model 1-2	Retrospective study	Between April 2008 and December 2022; Medical Data Vision database covering 485 acute care institutions; Hospitalized patients with cancer aged >18 years who received ICIs.
6	Zhihui Yan 2026 (40)	China	To investigate risk factors for cardiovascular toxicity following anti-PD-1/PD-L1 therapy and develop a predictive model.	Model 1	Two-center retrospective study	Between October 2018 and October 2023; Affiliated Hospital of Qingdao University and the Eighth People’s Hospital of Qingdao;Patients with solid tumors after receiving anti-PD-1/PD-L1 immunotherapy.
7	Yunhan Su 2026 (27)	China	To construct a risk prediction model for venous thromboembolism in lung cancer patients treated with ICIs.	Model 1	Single center retrospective study	Between January 2018 and March 2022; West China Hospital, Sichuan University; Lung cancer patients who received ICIs.
8	Chadi Ayoub 2025 (29)	USA	To develop an artificial intelligence model to predict the increased likelihood for the development of ICI-related myocarditis and adverse cardiovascular events.	Model 1-3	Multicentre retrospective study	Between January 2011 and February 2022; At any of the 3 academic institution sites; Cancer patients treated with ICIs therapy.
9	Zhihui Yan 2025 (28)	China	To investigate risk factors for mortality due to cardiovascular toxicity after anti-PD-1/PD-L1 therapy and construct a corresponding prediction model.	Model 1	Single center retrospective study	Between October 2018 and October 2023; Affiliated Hospital of Qingdao University; Solid tumor patients with cardiovascular toxicity after anti-PD-1/PD-L1 immunotherapy.
10	Shanshan Li (1) 2025 (34)	China	To create a prediction model using cardiac biomarkers to identify patients prone to myocarditis during treatment.	Model 1	Single center retrospective study	Between June 2018 and August 2024; Shandong Cancer Hospital and Institute, Shandong First Medical University; Patients with advanced or metastatic lung cancer received PD-1 or PD-L1 inhibitors as monotherapy or combination therapy.
11	Shanshan Li (2) 2025 (24)	China	To integrate multi-dimensional indicators to develop prediction models for identifying high-risk populations of ICI-related myocarditis.	Model 1-5	Single center retrospective study	Between June 2018 and August 2024; Shandong Cancer Hospital and Institute, Shandong First Medical University; patients with locally advanced or metastatic lung cancer who received immunotherapy and presented abnormal baseline cardiac parameters.
12	Zhengkun Guan 2025 (32)	China	To develop nomogram prognostic models for both short-term and long-term survival outcomes in patients with ICI myocarditis based on key biomarkers in peripheral blood.	Model 1-2	Single center retrospective study	Between January 2019 and June 2024; Fourth Hospital of Hebei Medical University; Patients who received immunotherapy and diagnosed with ICI myocarditis.
13	Jialian Li 2025 (19)	China	To develop an interpretable model that leverages readily available clinical data to predict early cardiotoxicity.	Model 1-3	Single center retrospective study	Between January 2020 and December 2023; First Affiliated Hospital of Chongqing Medical University; Patients who received their first ICI treatment.
14	Xiaotong Xia 2025 (38)	China	To develop a scoring system tailored for Asian populations through quantifying venous thromboembolism risk in a cohort of hospitalized cancer patients receiving ICIs.	Model 1	Single center retrospective study	Between January 2022 and June 2024; Zhongshan Hospital (Xiamen), Fudan University; Patients treated with ICIs.
15	Xiaoyun Cheng 2025 (30)	China	To identify risk factors and prognostic indicators, develop and evaluate a survival prediction model specific to ICI-related myocarditis, and explore diagnostic strategies and treatment management approaches.	Model 1	Multicentre retrospective study	Between January 2019 and December 2022 (training set) and December 2022 to November 2023 (external validation set); Four large tertiary care medical centers; Lung cancer patients with ICI-related myocarditis.
16	Qiuyue Li 2024 (23)	China	To analyze the incidence, high-risk factors of immunotherapy-related cardiotoxicity in non-small cell lung cancer patients and construct a risk prediction model.	Model 1	Single center observational study	Between October 2022 and December 2023; Cancer Hospital, Chinese Academy of Medical Sciences; Non-small cell lung cancer patients receiving immunotherapy-based medication regimens.
17	Yihuai Hu 2024 (22)	China	To compare and evaluate the risk factors for venous thromboembolism during immunotherapy in patients with non-small cell lung cancer, and construct a nomogram model.	Model 1	Single center retrospective study	Training set: Between September 2020 and August 2022; Affiliated Cancer Hospital of Guangzhou Medical University; Non-Small Cell Lung Cancer patients treated with ICIs. External validation set: Between September 2022 and August 2023; Affiliated Cancer Hospital of Guangzhou Medical University; Non-Small Cell Lung Cancer patients treated with ICIs.
18	Zhenli Li 2024 (35)	China	To establish nomograms based on ratio biomarkers to predict the severity and prognosis of ICI-related myocarditis.	Model 1-2	Single center retrospective study	From January 2020 to December 2023; Fourth Hospital of Hebei Medical University; Patients with a pathologic diagnosis of advanced cancers who received at least one cycle of ICI therapy.
19	Guanzhong Liang 2024 (36)	China	To develop and validate a nomogram model for predicting the occurrence of venous thromboembolism in lung cancer patients undergoing ICI therapy.	Model 1	Single center retrospective study	Between 2019 and 2022; Chongqing University Cancer Hospital; Lung cancer patients undergoing ICI inpatient treatment.
20	Jiarui Zhang 2024 (41)	China	To validate and compare the performances of the Caprini, Padua and Khorana risk scores in lung cancer patients receiving ICIs.	Model 1-3	Single center retrospective study	Between January 2018 and March 2022; West China Hospital; Lung patients received at least one dose of an approved ICIs.
21	Rui Lu 2024 (25)	China	To explore the risk factors for major adverse cardiovascular events in patients with ICI-associated myocarditis; develop a prediction model for MACE based on clinical data, electrocardiogram findings and echocardiographic characteristics and preliminarily explores the application of machine learning algorithms in predicting MACE among patients with ICI-related myocarditis.	Model 1-5	Single center retrospective study	Between May 2019 and August 2023; First Affiliated Hospital of Guangzhou Medical University; Tumor patients with ICI-related myocarditis.
22	Xitong Cheng 2024 (31)	China	To develop a prediction and grading model for immune-related cardiac adverse events, enabling graded management of patients.	Model 1-44	Two-center retrospective study	Between January 2011 and August 2022; Two medical institutions; Cancer patients treated with PD-1/PD-L1 Inhibitors.
23	Samuel Peter Heilbroner 2021 (33)	USA	To create a machine learning model to predict cardiac events in patients treated with ICIs.	Model 1	Retrospective study	Between 2013 and 2019; CancerLinQ database curated by the American Society of Clinical Oncology; Patients taking PD-1 or PD-L1 therapy.

ICI, immune checkpoint inhibitor; PD-1, programmed cell death protein 1; PD-L1, programmed death-ligand 1.

### 3.2 Model characteristics

These 23 studies included 84 prediction models. Eighty-two models were prognostic prediction models. Among them, 71 were constructed to predict the occurrence risk of ICI-related cardiovascular toxicities [53 for overall ICI-related cardiovascular toxicities/severe cardiovascular toxicities (19, 23, 29, 31, 33, 40), 11 for ICI-related myocarditis/severe myocarditis (24, 26, 34, 37, 39, 42), and seven for ICI-related venous thromboembolism (22, 27, 36, 38, 41)]. The remaining 11 models predicted the prognosis of patients with ICI-related cardiovascular toxicities [four for mortality risk (28, 30, 32) and seven for major adverse cardiovascular/cardiomyotoxic event (MACE) risk following the onset of ICI-related cardiovascular toxicity (15, 25, 35)]. Two were diagnostic prediction models for grading the severity of ICI-related myocarditis (35, 42).

#### 3.2.1 Characteristics of prediction model for the occurrence risk of overall ICI-related cardiovascular toxicity

All 53 models in six studies predicting the occurrence risk of overall ICI-related cardiovascular toxicity (including 22 models for severe ICI-related cardiovascular toxicity) were newly developed (see **Supplementary Table 1-1** for prediction outcome diagnostic criteria/definitions). Three models in one study (19) had a 30-day prediction time frame post-ICI treatment, one model in one study (23) covered three treatment cycles (21 days per cycle), and 49 models in four studies (29, 31, 33, 40) lacked a clearly reported time frame. Candidate predictors included patients’ demographic characteristics, medical history, treatment-related characteristics, and laboratory and imaging parameters. The number of final predictors ranged from four to 356. Common predictors in models included B-type natriuretic peptide (eight times), age (five times), cardiac troponin (four times), C-reactive protein (four times), creatine kinase (four times), and interleukin-2 (four times) (see **Supplementary Tables 1-1**, **2-1** for candidate predictors and final predictors). Two studies (four models) (29, 40) fully converted all continuous variables to categorical variables, one study (one model) (23) partially discretized continuous variables, one study (one model) (33) failed to specify the handling of continuous variables, and the remaining two studies (47 models) (19, 31) retained continuous variables in their original form. Two studies (four models) (29, 40) did not clearly report predictors collection time, one study (44 models) (31) collected predictors one month before patients received treatment, one study (one model) (33) collected predictors six months before ICI treatment or 15/30 days post-ICI treatment, and the remaining two studies (four models) (19, 23) collected predictors before initiation of ICI therapy without a specific time. Three studies (46 models) (23, 31, 40) had an events-per-variable (EPV) <10 during the model development stage, and others failed to clearly report EPV. For predictors selection, three studies (25 models) (19, 23, 31) used only univariate and/or multivariate analyses, one study (one model) (40) combined these statistical methods with clinical experience or prior evidence, two studies (23 models) (19, 31) used feature importance analysis, and two studies (four models) (29, 33) did not report predictors screening approaches. Modeling approaches included logistic regression (23, 40) and machine learning algorithms (19, 29, 31, 33). Two studies (four models) (19, 40) did not report missing data handling approaches, one study (44 models) (31) applied multiple imputation combined with exclusion of severe missing variables without clearly specifying missing data causes or proportion, one study (one model) (33) adopted machine learning algorithm alongside sensitivity analysis by variables deletion or multiple imputation, one study (one model) (23) supplemented missing data during data collection, and one study (three models) (29) combined variable deletion for missing data and recoding missing data to one. Regarding model presentation, two models in two studies (23, 40) were presented with nomograms, three models in three studies (19, 29, 33) were interpreted using the SHapley Additive exPlanation (SHAP) method, and 48 models in three studies (19, 29, 31) lacked visual presentation. (see **Supplementary Table 3-1** for model characteristics).

#### 3.2.2 Characteristics of prediction models for the occurrence risk of ICI-related myocarditis

All 11 models in six studies predicting the occurrence risk of ICI-related myocarditis (including one medel for severe ICI-related myocarditis) were newly developed (see **Supplementary Table 1-2** for prediction outcome diagnostic criteria/definitions). Two models in one study (39) had a prediction time frame within three months after initiation of ICI treatment, and nine models in five studies (24, 26, 34, 37, 42) lacked a clearly reported time frame. Candidate predictors included patients’ demographic characteristics, medical history, treatment-related characteristics, and laboratory and imaging parameters. The number of final predictors ranged from three to 89. Common predictors in models included B-type natriuretic peptide (three times), cardiac troponin (three times), creatine kinase (two times), and α-hydroxybutyrate dehydrogenase (two times) (see **Supplementary Tables 1-2**, **2-2** for candidate predictors and final predictors). Two studies (two models) (34, 37) partially converted continuous variables to categorical variables, three studies (eight models) (24, 26, 39) retained continuous variables in their original form, and the remaining one study (one model) (42) failed to specify the handling of continuous variables. One study (two models) (39) did not clearly report predictors collection time, two studies (two models) (26, 37) collected predictors before both ICI initiation and ICI-associated myocarditis without specific time, one study (one model) (42) collected predictors before the initiation of ICI therapy without specific time and at the time of myocarditis diagnosis or only before the first ICI treatment without specific time, and the remaining two studies (six models) (24, 34) collected predictors before initiation of ICI therapy without specific time. Five studies (10 models) (24, 26, 34, 37, 39) had an EPV <10 during the model development stage, and one study (one model) (42) failed to clearly report EPV. For predictors selection, five studies (10 models) (24, 26, 34, 39, 42) used only univariate and/or multivariate analyses, and the remaining one study (one model) (37) combined univariate analyses with least absolute shrinkage and selection operator (LASSO) regression analysis. Modeling approaches included logistic regression (26, 34, 37, 42) and machine learning algorithms (24, 39). One study (one model) (34) did not report missing data handling approaches, and five studies (10 models) (24, 26, 37, 39, 42) enrolled patients with complete information. Regarding model presentation, four models in four studies (24, 26, 34, 37) were presented with nomograms, one model in one study (39) was interpreted using the SHAP method, and six models in three studies (24, 39, 42) lacked visual presentation. (see **Supplementary Table 3-2** for model characteristics).

#### 3.2.3 Characteristics of prediction model for the occurrence risk of ICI-related venous thromboembolism

Four models in four studies (22, 27, 36, 38) predicting the occurrence risk of ICI-related venous thromboembolism were newly developed. One study (41) externally validated three previously established models (see **Supplementary Table 1-3** for prediction outcome diagnostic criteria/definitions). Five models in three studies (27, 38, 41) had a one-year prediction time frame post-ICI treatment, and two models in two studies (22, 36) lacked a clearly reported time frame. Candidate predictors in newly developed models included patients’ demographic characteristics, medical history, treatment-related characteristics, and laboratory parameters. The number of final predictors in newly developed models ranged from five to 10. Common predictors in newly developed models included chemotherapy (two times), D-dimer (two times), Eastern Cooperative Oncology Group performance status (two times), and smoking (two times) (see **Supplementary Tables 1-3**, **2–3** for candidate predictors and final predictors). For newly developed models, three studies (three models) (22, 27, 36) partially converted continuous variables to categorical variables and one study (one model) (38) failed to specify the handling of continuous variables. Four studies (six models) (27, 36, 38, 41) did not clearly report predictors collection time, and one study (one model) (22) collected predictors before initiation of ICI therapy without a specific time. For newly developed models, three studies (three models) (22, 27, 36) had an EPV <10 during the model development stage and one study (one model) (38) failed to clearly report EPV. For the study (41) externally validating three previously established models, both event and non-event counts exceeded 100. For predictors selection in newly developed models, all four studies (four models) (22, 27, 36, 38) used only univariate and multivariate analyses. Modeling approaches were logistic regression in newly developed models (22, 27, 36, 38). Three studies (five models) (27, 38, 41) did not report missing data handling approaches, one study (one model) (22) applied random forest imputation without clearly specifying the causes or proportion of missing data, and one study (one model) (36) enrolled patients with complete information. Regarding model presentation, two models in two studies (22, 36) were presented with nomograms and five models in three studies (27, 38, 41) were presented with a scoring system. (see **Supplementary Table 3-3** for study characteristics).

#### 3.2.4 Characteristics of prediction model for the risk of mortality following ICI-related cardiovascular toxicity

All four models in three studies predicting the risk of mortality following ICI-related cardiovascular toxicity were newly developed (see **Supplementary Table 1-4** for prediction outcome diagnostic criteria/definitions). One model in one study (28) predicted the 120-day mortality risk following the onset of overall ICI-related cardiovascular toxicity, one model in one study (30) predicted mortality risk from 0.5 to 1.5 years following the onset of myocarditis, one model in one study (32) predicted 40-day mortality risk following the onset of ICI-related myocarditis, and one model in one study (32) predicted the one-year mortality risk following the onset of ICI-associated myocarditis. Candidate predictors included patients’ demographic characteristics, medical history, treatment-related characteristics, and laboratory and imaging parameters. The number of final predictors ranged from three to four. Common predictors in models included cardiac troponin (two times), lactic dehydrogenase-to-albumin ratio (two times), and B-type natriuretic peptide (two times) (see **Supplementary Tables 1-4**, **2-4** for candidate predictors and final predictors). One study (one model) (30) partially converted continuous variables to categorical variables, and two studies (three models) (28, 32) retained continuous variables in their original form. All three studies (four models) (28, 30, 32) did not clearly report predictors collection time. All three studies (four models) (28, 30, 32) had an EPV <10 during the model development stage. For predictors selection, one study (one model) (28) combined univariate and multivariate analyses with prior evidence and two studies (three models) (30, 32) combined univariate analyses with LASSO regression analysis. Modeling approaches were Cox regression analysis (28, 30, 32). One study (one model) (28) did not report missing data handling approaches, and two studies (three models) (30, 32) enrolled patients with complete information. Regarding model presentation, two models in two studies (28, 30) were presented with nomograms and two models in one study (32) were presented with nomograms and a web-based application. (see **Supplementary Table 3-4** for model characteristics).

#### 3.2.5 Characteristics of prediction model for the risk of MACE following ICI-related cardiovascular toxicity

All seven models in three studies predicting the occurrence risk of MACE following ICI-related cardiovascular toxicity were newly developed (see **Supplementary Table 1-5** for prediction outcome diagnostic criteria/definitions). One model in one study (15) had a 30-day prediction time frame following ICI-related myocarditis, one model in one study (35) had a 40-day prediction time frame following ICI-related myocarditis, and five models in one study (25) lacked a clearly reported time frame following ICI-associated myocarditis. Candidate predictors included patients’ demographic characteristics, medical history, treatment-related characteristics, and laboratory and imaging parameters. The number of final predictors ranged from two to six. Common predictors in models included left ventricular ejection fraction (two times), neutrophil-to-lymphocyte ratio (two times), and ST-T segment changes (two times) (see **Supplementary Tables 1-5**, **2-5** for candidate predictors and final predictors). Two studies (six models) (15, 25) partially converted continuous variables to categorical variables, and another study (one model) (35) retained continuous variables in their original form. Two studies (six models) (15, 25) did not clearly report predictors collection time, and one study (one model) (35) collected predictors before initiation of ICI therapy without specific time and at the onset of myocarditis. One study (one model) (15) had an EPV <10 during the model development stage, and others (25, 35) failed to clearly report EPV. For predictors selection, one study (five models) (25) used only univariate and/or multivariate analyses, one study (one model) (15) combined these statistical methods with clinical experience or prior evidence, and one study (one model) (35) combined univariate/multivariate analysis with LASSO regression analysis. Modeling approaches included logistic regression analysis (25), Cox regression analysis (15, 35), and machine learning algorithms (25). One study (one model) (35) did not report missing data handling approaches, one study (one model) (15) applied multiple imputation without clearly specifying the causes or proportion of missing data, and one study (five models) (25) enrolled patients with complete information. Regarding model presentation, one model in one study (15) was presented with a risk scoring system, one model in one study (35) was presented with a nomogram and web-based application, one model in one study (25) was presented with a nomogram and regression formula, one model in one study (25) was interpreted using the SHAP method, and three models in one study (25) lacked visual presentation. (see **Supplementary Table 3-5** for model characteristics).

#### 3.2.6 Characteristics of diagnostic prediction model for grading the severity of ICI-related cardiovascular toxicity

All two models in two studies (35, 42) for grading the severity of ICI-related cardiovascular toxicity were newly developed, and the prediction outcomes were ICI-associated myocarditis (see **Supplementary Table 1-6** for prediction outcome diagnostic criteria/definitions). Candidate predictors included patients’ demographic characteristics, medical history, treatment-related characteristics, and laboratory parameters. The number of final predictors ranged from two to three. Final predictors in models included cardiac and laboratory parameters (see **Supplementary Tables 1-6** and **2-6** for candidate predictors and final predictors). One study (one model) (35) retained continuous variables in their original form, and one study (one model) (42) failed to specify the handling of continuous variables. Two studies (two models) (35, 42) collected predictors before initiation of ICI therapy without a specific time and at the onset of myocarditis. One study (one model) (35) had an EPV <10 during the model development stage, and another (42) failed to clearly report EPV. For predictors selection, two studies (two models) (35, 42) used only univariate and multivariate analyses. Modeling approaches were logistic regression analysis (35, 42). One study (one model) (35) did not report missing data handling approaches, and one study (one model) (42) enrolled patients with complete information. Regarding model presentation, one model in one study (35) was presented with a nomogram and web-based application and one model in one study (42) lacked visual presentation. (see **Supplementary Table 3-6** for model characteristics).

### 3.3 Validation and performance of prediction model

#### 3.3.1 Performance of prediction model for the occurrence risk of overall ICI-related cardiovascular toxicity

One study (one model) (23) assessed apparent performance. The model exhibited acceptable discrimination performance, with an area under the receiver operating characteristic curve (AUC) of 0.935. Calibration performance of the model was also acceptable (supported by well-fitted calibration curves and a favorable Hosmer–Lemeshow test). Decision curve analysis (DCA) revealed that the model yielded a wide range of clinical net benefit.

Five studies (52 models) conducted internal validation, using bootstrap (40), splitting (29, 33), cross-validation (31), and splitting + bootstrap (19) methods. The discrimination performance of the 52 models was assessed. Forty-four models (19, 29, 31, 40) had acceptable discrimination performance (AUC: 0.7091–0.93). Two models demonstrated unstable discrimination performance (one had a bootstrap-adjusted AUC of 0.60 in the training set and 0.80 in the test set (19), and the AUC of the another fluctuated between 0.63 and 0.72, with varying predictive time windows (33)). Six models (29, 31) demonstrated unsatisfactory discrimination performance (AUC <0.7). Calibration performance of two models (19, 40) was assessed and deemed acceptable (supported by well-fitted calibration curves and/or a favorable Hosmer–Lemeshow test). Clinical utility was assessed in four models (19, 40) (DCA and/or clinical impact curve (CIC) analysis demonstrating broad net benefits). The overall performance of 44 models (31) was demonstrated via a Brier score within the range of 0.0846–0.3352. The classification performance of 51 models (19, 29, 31, 40) was assessed. (see **Supplementary Table 4-1** for model performance).

#### 3.3.2 Performance of prediction model for the occurrence risk of ICI-related myocarditis

Four studies (eight models) (24, 34, 37, 42) assessed apparent performance. The discrimination performance of three models (34, 37, 42) was assessed. Two (34, 37) had acceptable discrimination performance (AUC: 0.831–0.926), and one (42) had unsatisfactory discrimination performance (AUC <0.7). Calibration performance of one model (37) was assessed and deemed acceptable (supported by well-fitted calibration curves). Clinical utility was assessed in one model (37) (DCA demonstrating broad net benefits). The classification performance of six models (24, 34) was assessed.

Six studies (11 models) conducted internal validation, using bootstrap (42), splitting (24, 34, 37), splitting+bootstrap (26) and splitting+cross validation (39) methods. The discrimination performance of 11 models was assessed. Eight models (24, 26, 34, 37) exhibited acceptable discrimination performance (AUC: 0.844–0.942), and three (39, 42) demonstrated unsatisfactory discrimination performance (AUC < 0.7). Calibration performance of four models (24, 26, 34, 37) was assessed and deemed acceptable (supported by well-fitted calibration curves and/or a favorable Hosmer–Lemeshow test). Clinical utility was assessed in two models (26, 37) (DCA demonstrating broad net benefits). The classification performance of seven models (24, 39) was assessed.

One study (one model) (37) performed external validation. The model exhibited acceptable discrimination performance (AUC: 0.949). The calibration performance and clinical utility of the model were acceptable (supported by calibration curves and DCA, respectively). (see **Supplementary Table 4-2** for model performance).

#### 3.3.3 Performance of prediction model for the occurrence risk of ICI-related venous thromboembolism

Three studies (three models) (22, 27, 36) assessed apparent performance. The three models exhibited acceptable discrimination performance (AUC: 0.815–0.828). Calibration performance of two models (27, 36) were assessed and deemed acceptable (supported by well-fitted calibration curves and/or a favorable Hosmer–Lemeshow test). Clinical utility was assessed in two models (22, 36) (DCA and/or CIC analysis demonstrating broad net benefits). The overall performance of one model (36) was confirmed by a Brier score of 0.068. The classification performance of one model (22) was assessed.

Four studies (four models) conducted internal validation, using bootstrap (22) and splitting (27, 36, 38) methods. Discrimination performance of three models (27, 36, 38) was assessed and deemed acceptable (AUC: 0.753–0.818). Calibration performance of three models (22, 27, 36) was assessed and deemed acceptable (supported by well-fitted calibration curves and/or a favorable Hosmer–Lemeshow test). Clinical utility was assessed in one model (36) (DCA and CIC analysis demonstrating broad net benefits). The overall performance of one model (36) was demonstrated via a Brier score of 0.080.

Two studies (four models) (22, 41) performed external validation. The discrimination performance of the four models was assessed. Two models (22, 41) had acceptable discrimination performance (AUC: 0.743–0.794), one had (41) unstable discrimination performance (AUC: 0.656–0.745 across different patient groups), and one (41) had unsatisfactory discrimination performance (AUC < 0.7). Calibration performance and clinical utility were assessed in one model (22) and exhibited acceptable calibration performance and clinical utility (supported by calibration curves and DCA, respectively). (see **Supplementary Table 4-3** for model performance).

#### 3.3.4 Performance of prediction model for the risk of mortality following ICI-related cardiovascular toxicity

Two studies (three models) (28, 32) assessed apparent performance. The three models exhibited acceptable discrimination performance (AUC: 0.765–0.880; C statistic: 0.742–0.824). Calibration performance of one model (28) was assessed and deemed acceptable (supported by well-fitted calibration curves). Clinical utility was assessed in the three models (28, 32) (DCA demonstrating broad net benefits). The overall performance of two models (32) was demonstrated via a Brier score within the range of 0.12–0.18.

Two studies (three models) conducted internal validation, using bootstrap methods (30, 32). The three models exhibited acceptable discrimination performance (AUC: 0.832–0.924; C statistic: 0.728–0.812). The three models also exhibited acceptable calibration performance (supported by well-fitted calibration curves). Clinical utility was assessed in one model (30) (DCA demonstrating broad net benefits).

One study (one model) (30) performed external validation. The model exhibited acceptable discrimination performance (the time-dependent AUC:0.789-0.821), as well as calibration performance and clinical utility (supported by calibration curves and DCA, respectively). (see **Supplementary Table 4-4** for model performance).

#### 3.3.5 Performance of prediction model for the risk of MACE following ICI-related cardiovascular toxicity

Two studies (two models) (25, 35) assessed apparent performance. The two models exhibited acceptable discrimination performance (AUC: 0.967; C statistic:0.807). The classification performance of one model (25) was assessed.

Three studies (six models) conducted internal validation, using bootstrap (35) and cross-validation methods (15, 25). The six models exhibited acceptable discrimination performance (AUC: 0.784–0.892; C statistic: 0.70–0.779). The calibration performance of five models (25, 35) was assessed, and three (25, 35) exhibited acceptable calibration performance (supported by well-fitted calibration curves). The clinical utility of one model (35) was assessed (DCA demonstrating broad net benefits). The classification performance of four models (25) was assessed.

One study (one model) (15) performed external validation. The model exhibited unstable discrimination performance (time–dependent C statistic 0.63–0.75). (see **Supplementary Table 4-5** for model performance).

#### 3.3.6 Performance of diagnostic prediction model for grading the severity of ICI-related cardiovascular toxicity

Two studies (two models) (35, 42) assessed apparent performance. The two models exhibited acceptable discrimination performance (AUC: 0.769, C statistic: 0.757).

Two studies (2 models) conducted internal validation using the bootstrap methods (35, 42). One model (35) exhibited acceptable discrimination performance (C statistic: 0.752), as well as calibration performance and clinical utility (supported by well-fitted calibration curves and DCA demonstrating broad net benefits). (see **Supplementary Table 4-6** for model performance).

### 3.4 Quality, risk of bias, and applicability assessment

For prediction models that predict the occurrence risk of overall ICI-related cardiovascular toxicity, all models (19, 23, 29, 31, 33, 40) included exhibited high-quality problems and risk of bias, and only four models (19, 23) exhibited high applicability in the model development and validation stage. For prediction models that predict the occurrence risk of ICI-related myocarditis, the occurrence risk of ICI-related venous thromboembolism, and the risk of mortality following ICI-related cardiovascular toxicity, all models (22, 24, 26–28, 30, 32, 34, 36–39, 41, 42) included exhibited high-quality problems, risk of bias, and unclear applicability in the model development and validation stage. For prediction models predicting the risk of MACE following ICI-related cardiovascular toxicity and grading the severity of ICI-related cardiovascular toxicity, all models (15, 25, 35, 42) included exhibited high quality problem and risk of bias, with only one model (35) and two models (35, 42) exhibiting high applicability respectively in the model development and validation stage. (see **Supplementary Figures 1-1~1-6** for quality, risks of bias and applicability assessment).

## 4 Discussion

We systematically reviewed prognostic prediction models that predict the occurrence risk and prognosis of ICI-related cardiovascular toxicity and diagnostic prediction models that grade the severity of such toxicity. The discrimination performance of all models was assessed using apparent, internal, and/or external performance validation, with the majority exhibiting acceptable performance. Partial models exhibited acceptable calibration performance and clinical utility. However, all models exhibited high quality problem and risk of bias, and most models exhibited unclear applicability in the model development and validation stage. This indicates that these models require further validation and improvement before being widely applied in clinical practice.

### 4.1 Current models exhibited high quality problem and risk of bias

This study found that all models exhibited high quality problem and risk of bias regardless of the type of prediction models, consistent with the current status of prediction model studies (43, 44, 50). This can be explained as follows: First, in the participants and data sources domain, except for one model (23), all others were based on retrospective studies and some (15, 33, 39) used data from public or subscription-based databases. Although this reduced research costs, it may lead to inaccurate evaluation of predictors and outcomes (51). Second, in the predictors domain, only one model (23) based on a prospective design inherently ensured blinding between predictors collection and outcomes assessment; other models based on retrospective designs did not report blinding adoption. Lack of blinding may increase assessment bias, especially for subjectively judged predictors (48). Additionally, some studies (15, 25, 27–30, 32, 33, 36, 38–41) did not clearly state predictors and/or predictors collection time, rendering it difficult to determine predictors availability during model application (48). Third, in the outcome domain, no model reported blinding between outcomes assessment and predictors collection, risking information leakage between outcomes and predictors. Moreover, some studies (15, 22, 24–34, 36–42) did not clearly report outcomes evaluation time or predictors collection time, affecting the judgment of the rationality of the interval between the outcomes evaluation and predictors collection (48). Fourth, in the analysis domain, during the model development stage for binary outcomes, sample size is usually determined based on EPV. To prevent model overfitting, the EPV is usually required to be at least greater than 10 (49). In the model validation stage, the number of event and non-event cases should generally be at least 100 (49). However, most models included failed to meet these sample size criteria. Some studies (15, 22, 23, 25, 27, 29, 30, 34, 36, 37, 40) categorized continuous variables using variable processing methods, potentially causing information loss (48, 52). Proper reporting and handling of missing data are essential, as most missing data are non-random and may cause bias in model predictive performance (49, 53, 54). However, all studies did not explicitly report missing data information, and some (24–26, 29–32, 36, 37, 39, 42) excluded patients with incomplete information. Methods for mitigating overfitting include selecting an adequate sample size relative to model complexity, avoiding purely data-driven predictors selection, and applying regularization/shrinkage methods (48, 49, 52). However, some studies (19, 22–27, 31, 34–36, 38, 42) used only univariate/multivariate analyses for predictors selection without regularization/shrinkage methods. Even models (30, 32, 35, 37) using LASSO regression employed small sample sizes, failing to fully reduce overfitting risk. For model validation, insufficient performance validation (lack of discrimination/calibration) and over-reliance on apparent validation for calibration are problematic (48). However, several models (15, 19, 24, 25, 29, 31, 33, 38, 39, 41, 42) lacked calibration validation, and several others (23, 28) only underwent apparent calibration validation.

### 4.2 Current prediction models have limited clinical applicability

The clinical application of current prediction models is restricted. First, some models (15, 22, 24–34, 36–42) failed to clearly specify the time points for predictors collection and outcomes evaluation, and partial models (29, 33, 39) had unclear final predictors, resulting in unclear applicability assessment results. Second, all models exhibited high quality problems and risk of bias, which further hindered their clinical application. Notably, in most newly constructed prediction models, the majority had a small EPV value. While the reported predictive performance of these models seems satisfactory, these estimates are prone to instability owing to overfitting, especially among models with EPV < 5. Third, the validation of model performance was inadequate. Sufficient external validation is a prerequisite for the widespread clinical application of prediction models (44, 49, 55). In this review, only seven models (15, 22, 30, 37, 41) were externally validated, and most validation samples were small. Accordingly, inadequate external validation is another restricting factor. In addition, several models lacked calibration validation, and several others underwent only apparent calibration validation. Fourth, in addition to the unclear final predictors in partial models, some models (29, 33, 39) included an excessive number of final predictors, increasing the difficulty and cost of clinical application. Finally, the absence of visualization presentation in partial models (19, 24, 25, 29, 31, 39, 42) is not conducive to clinical use.

### 4.3 Current prediction models exhibited high heterogeneity

Various types of prediction models for ICI-related cardiovascular toxicity have been reported. However, certain heterogeneity exists among models of the same type, which is mainly manifested in the following aspects. First, heterogeneity exists in the prediction outcome diagnostic criteria or definitions. For example, regarding overall ICI-related cardiovascular toxicity, some studies (19, 23) defined it as abnormalities in some indicators such as electrocardiograms and myocardial markers, whereas others (29, 33, 40) defined it as specific subtypes of heart diseases such as myocarditis and arrhythmia. Moreover, the referenced diagnostic criteria exhibit heterogeneity, with some studies (31, 40) adopting the 2022 ESC Guidelines on Cardio-Oncology, some (23) using the Guidelines on Cardio-Oncology from the Chinese Society of Clinical Oncology (CSCO), and some (19) referring to the National Comprehensive Cancer Network Guidelines. Similarly, for ICI-related myocarditis, some studies (24, 26, 34, 42) referred to the 2022 ESC Guidelines on Cardio-Oncology as the diagnostic criteria and 2025 ESC Guidelines for the management of myocarditis and pericarditis, and some (39) adopted the International Classification of Diseases (ICD) for diagnosis. For the risk of mortality following ICI-related cardiovascular toxicity, some studies (30, 32) specifically focused on the risk of mortality following ICI-related myocarditis, whereas some (28) targeted the risk of mortality from overall ICI-related cardiovascular toxicity. For MACE, one study (15) included respiratory failure due to myositis-related respiratory muscle failure requiring ventilation in the definition of MACE, whereas others did not include this condition. For the grading standards for severity of ICI-related cardiovascular toxicity, some studies (31, 37, 42) adopted version 5 of the Common Terminology Criteria for Adverse Events and some (35) used version 4. Second, heterogeneity is observed in the prediction time frame. For instance, among models predicting the occurrence risk of overall ICI-related cardiovascular toxicity, some (19) set the prediction time frame to 30 days, whereas some (23) defined it as three treatment cycles. Among models predicting the risk of mortality following ICI-related cardiovascular toxicity, some (28) set the prediction time frame to 120 days, whereas others (30, 32) set it to 40 days, 1 year, or 0.5–1 year. Among models predicting the risk of MACE following ICI-related cardiovascular toxicity, some (15) set the prediction time frame to 30 days, whereas some (35) set it to 40 days. Third, although candidate/final predictors mainly include demographic variables, medical history, treatment-related variables, and laboratory and imaging parameters, differences were observed in the candidate and final predictors among similar models. Considering the timing of predictors assessment, some studies (31, 33) collected predictors 1 or 6 months before treatment, whereas others (19, 22, 24, 26, 34, 35, 37, 42) reported that the predictors assessment time was before treatment, without specifying the exact time. Fourth, for modeling methods with similar models, some adopted conventional regression methods, whereas others used machine learning approaches. Fifth, some models validated only apparent, internal, or external performance, whereas others validated two to three of these types of performance simultaneously. In addition, some models evaluated only discrimination performance, whereas others evaluated calibration performance and clinical utility. Finally, for study populations, some studies (22–24, 27, 30, 34, 36, 37, 41) only included lung cancer patients, whereas others (15, 19, 25, 26, 28, 29, 31–33, 35, 38–40, 42) included various cancer patients. This heterogeneity hinders the comparison between similar models, rendering it difficult to determine the best model, and increases the difficulty of clinical selection and application.

## 5 Implications for future research

First, to improve methodological quality and reduce risk of bias in model development and validation stages, studies should comply with relevant standards or appraisal tools such as TRIPOD standards and the PROBAST+AI tool in future studies (47, 48, 56). Specifically, it is imperative to strictly adhere to sample size-related requirements and clearly report predictors incorporated into the model and their assessment time, whether blinding was implemented between predictors and outcomes assessment, as well as the prediction time frame of the model. Meanwhile, the handling of missing data and continuous variables, strategies adopted to mitigate model overfitting, and adequate and rigorous model validation should be considered. Second, to advance clinical translation of existing models, researchers should further perform large-sample multi-center external validation studies and optimize these models. Third, to reduce model heterogeneity, researchers may adopt core outcome set research ideas (57, 58). Combining expert consensus, patient needs, clinical evidence, and real-world experience, researchers can sort out patients’ demographic, therapeutic, laboratory, and imaging indicators and establish a unified core predictors set for evaluating the occurrence, prognosis, and severity of immunotherapy-related cardiovascular toxicity. Standard definitions and assessment criteria for predictors and outcomes should also be formulated. Additionally, the optimal prediction time frame and predictors assessment time (such as fixed time nodes or dynamic assessment nodes), as well as model modeling methods, should be explored and determined using consensus- or evidence-based methods. Finally, large-sample multi-center designs are suggested for model development and validation to strengthen model comparability and external applicability.

## 6 Strengths and limitations

To the best of our knowledge, this is the first comprehensive systematic review focusing on prediction models for ICI-related cardiovascular toxicity. We conducted a systematic literature search across multiple databases and used the updated PROBAST+AI tools for quality, risk of bias, and applicability assessment.

This study has some limitations. First, only English and Chinese literature were included, which may lead to missing relevant studies. Second, most models were developed and validated in China, restricting their geographical generalizability. Third, the substantial heterogeneity across the included models precluded the quantitative pooled analysis of their predictive performance.

## 7 Summary

This study systematically assessed prediction models for the occurrence, prognosis, and severity of ICI-related cardiovascular toxicity. All models had high-quality problems and risk of bias, and most exhibited unclear applicability. Accordingly, prior to further adequate validation and optimization of existing models, their widespread application in clinical practice is not recommended. Under current circumstances, baseline and dynamic risk screening using relevant risk biomarkers is suggested to better manage ICI-related cardiovascular toxicity. Future studies should follow rigorous methods to develop new models or conduct further validation and optimization of existing models.

## Data Availability

The original contributions presented in the study are included in the article/**Supplementary Material**. Further inquiries can be directed to the corresponding author.
