# Supplementary material for: Prediction models for immune checkpoint inhibitor-related cardiovascular toxicity: a systematic review
Source: Front Immunol. 2026 Aug 19;17:1903131. doi: 10.3389/fimmu.2026.1903131 (PMC13534096; doi:10.3389/fimmu.2026.1903131)
Supplement: Supplementary file 1 [file Table1.docx]

Supplementary Material

Supplementary Material Contents

Document: Literature Search Strategy

Table 1-1 Candidate predictors and outcome diagnostic criteria/definitions of prediction model for the occurrence risk of overall immune checkpoint inhibitor-related cardiovascular toxicity

Table 1-2 Candidate predictors and outcome diagnostic criteria/definitions of prediction model for the occurrence risk of immune checkpoint inhibitor-related myocarditis

Table 1-3 Candidate predictors and outcome diagnostic criteria/definitions of prediction model for the occurrence risk of immune checkpoint inhibitor-related venous thromboembolism

Table 1-4 Candidate predictors and outcome diagnostic criteria/definitions of prediction model for the risk of mortality following immune checkpoint inhibitor-related cardiovascular toxicity

Table 1-5 Candidate predictors and outcome diagnostic criteria/definitions of prediction model for the risk of major adverse cardiovascular/cardiac events following immune checkpoint inhibitor-related cardiovascular toxicity

Table 1-6 Candidate predictors and outcome diagnostic criteria/definitions of diagnostic prediction model for grading the severity of immune checkpoint inhibitor-related cardiovascular toxicity

Table 2-1 Final predictors of prediction model for the occurrence risk of overall immune checkpoint inhibitor-related cardiovascular toxicity

Table 2-2 Final predictors of prediction model for the occurrence risk of immune checkpoint inhibitor-related myocarditis

Table 2-3 Final predictors of prediction model for the occurrence risk of immune checkpoint inhibitor-related venous thromboembolism

Table 2-4 Final predictors of prediction model for the risk of mortality following immune checkpoint inhibitor-related cardiovascular toxicity

Table 2-5 Final predictors of prediction model for the risk of major adverse cardiovascular/cardiac events following immune checkpoint inhibitor-related cardiovascular toxicity

Table 2-6 Final predictors of diagnostic prediction model for grading the severity of immune checkpoint inhibitor-related cardiovascular toxicity

Table 3-1 Characteristics of prediction model for the occurrence risk of overall immune checkpoint inhibitor-related cardiovascular toxicity

Table 3-2 Characteristics of prediction model for the occurrence risk of immune checkpoint inhibitor-related myocarditis

Table 3-3 Characteristics of prediction model for the occurrence risk of immune checkpoint inhibitor-related venous thromboembolism

Table 3-4 Characteristics of prediction model for the risk of mortality following immune checkpoint inhibitor-related cardiovascular toxicity

Table 3-5 Characteristics of prediction model for the risk of major adverse cardiovascular/cardiac events following immune checkpoint inhibitor-related cardiovascular toxicity

Table 3-6 Characteristics of diagnostic prediction model for grading the severity of immune checkpoint inhibitor-related cardiovascular toxicity

Table 4-1 Performance of prediction model for the occurrence risk of overall immune checkpoint inhibitor-related cardiovascular toxicity

Table 4-2 Performance of prediction model for the occurrence risk of immune checkpoint inhibitor-related myocarditis

Table 4-3 Performance of prediction model for the occurrence risk of immune checkpoint inhibitor-related venous thromboembolism

Table 4-4 Performance of prediction model for the risk of mortality following immune checkpoint inhibitor-related cardiovascular toxicity

Table 4-5 Performance of prediction model for the risk of major adverse cardiovascular/cardiac events following immune checkpoint inhibitor-related cardiovascular toxicity

Table 4-6 Performance of diagnostic prediction model for grading the severity of immune checkpoint inhibitor-related cardiovascular toxicity

Figure 1-1 Quality, risks of bias and applicability assessment of prediction model for the occurrence risk of overall immune checkpoint inhibitor-related cardiovascular toxicity

Figure 1-2 Quality, risks of bias and applicability assessment of prediction model for the occurrence risk of immune checkpoint inhibitor-related myocarditis

Figure 1-3 Quality, risks of bias and applicability assessment of prediction model for the occurrence risk of immune checkpoint inhibitor-related venous thromboembolism

Figure 1-4 Quality, risks of bias and applicability assessment of prediction model for the risk of mortality following immune checkpoint inhibitor-related cardiovascular toxicity

Figure 1-5 Quality, risks of bias and applicability assessment of prediction model for the risk of major adverse cardiovascular/cardiac events following immune checkpoint inhibitor-related cardiovascular toxicity

Figure 1-6 Quality, risks of bias and applicability assessment of diagnostic prediction model for grading the severity of immune checkpoint inhibitor-related cardiovascular toxicity

Document: Literature Search Strategy

China National Knowledge Infrastructure

SU=(预测模型 + 风险模型 + 预测工具 + 风险评分 + 风险评估 + 预测因子 + 生物标志物 + 模型 + 人工智能 + 机器学习 + 深度学习 + 列线图 + 诺莫图 + 区分度 + 校准度 + ROC曲线 + 决策曲线) AND SU=(免疫治疗 + 免疫检查点抑制剂 + 程序性死亡受体-1 + 程序性死亡配体-1 + 细胞毒性T淋巴细胞相关抗原-4 + PD-1 + PD-L1 + CTLA-4 + ICIs) AND SU=(免疫相关不良事件 + 不良反应 + 毒性 + 副作用 + irAEs + 症状 + 免疫治疗相关心脏毒性 + 免疫治疗相关心血管毒性 + 免疫治疗相关心肌炎 + 免疫检查点抑制剂相关心肌炎 + 免疫检查点抑制剂相关心脏毒性 + 免疫检查点抑制剂相关心血管毒性 + 静脉血栓 + 心律失常 + 心脏功能障碍 + 动脉粥样硬化 + 急性冠状动脉综合征 + 心包炎 + 血管炎 + 心肌炎 + 心脏不良事件 + 心血管不良事件 + 心脏毒性 + 心血管毒性)

Note: SU:subject terms; ROC:receiver operating curves; PD-1: Programmed cell death protein 1; PD-L1: Programmed death-ligand 1; CTLA-4: cytotoxic t-lymphocyte-associated antigen-4; ICIs: immune checkpoint inhibitors; irAEs: immune-related adverse events.

Wanfang Database

主题:(预测模型 OR 风险模型 OR 预测工具 OR 风险评分 OR 风险评估 OR 预测因子 OR 生物标志物 OR 模型 OR 人工智能 OR 机器学习 OR 深度学习 OR 列线图 OR 诺莫图 OR 区分度 OR 校准度 OR ROC曲线 OR 决策曲线) and 主题:(免疫治疗 OR 免疫检查点抑制剂 OR 程序性死亡受体-1 OR 程序性死亡配体-1 OR 细胞毒性T淋巴细胞相关抗原-4 OR PD-1 OR PD-L1 OR CTLA-4 OR ICIs) and 主题:(免疫相关不良事件 OR 不良反应 OR 毒性 OR 副作用 OR irAEs OR 症状 OR 免疫治疗相关心脏毒性 OR 免疫治疗相关心血管毒性 OR 免疫治疗相关心肌炎 OR 免疫检查点抑制剂相关心肌炎 OR 免疫检查点抑制剂相关心脏毒性 OR 免疫检查点抑制剂相关心血管毒性 OR 静脉血栓 OR 心律失常 OR 心脏功能障碍 OR 动脉粥样硬化 OR 急性冠状动脉综合征 OR 心包炎 OR 血管炎 OR 心肌炎 OR 心脏不良事件 OR 心血管不良事件 OR 心脏毒性 OR 心血管毒性)

Note: ROC:receiver operating curves; PD-1: Programmed cell death protein 1; PD-L1: Programmed death-ligand 1; CTLA-4: cytotoxic t-lymphocyte-associated antigen-4; ICIs: immune checkpoint inhibitors; irAEs: immune-related adverse events.

China Science and Technology Journal Database

题名或关键词：预测模型 OR 风险模型 OR 预测工具 OR 风险评分 OR 风险评估 OR 预测因子 OR 生物标志物 OR 模型 OR 人工智能 OR 机器学习 OR 深度学习 OR 列线图 OR 诺莫图 OR 区分度 OR 校准度 OR ROC曲线 OR 决策曲线

AND

题名或关键词：免疫治疗 OR 免疫检查点抑制剂 OR 程序性死亡受体-1 OR 程序性死亡配体-1 OR 细胞毒性T淋巴细胞相关抗原-4 OR PD-1 OR PD-L1 OR CTLA-4 OR ICIs

AND

题名或关键词：免疫相关不良事件 OR 不良反应 OR 毒性 OR 副作用 OR irAEs OR 症状 OR 免疫治疗相关心脏毒性 OR 免疫治疗相关心血管毒性 OR 免疫治疗相关心肌炎 OR 免疫检查点抑制剂相关心肌炎 OR 免疫检查点抑制剂相关心脏毒性 OR 免疫检查点抑制剂相关心血管毒性 OR 静脉血栓 OR 心律失常 OR 心脏功能障碍 OR 动脉粥样硬化 OR 急性冠状动脉综合征 OR 心包炎 OR 血管炎 OR 心肌炎 OR 心脏不良事件 OR 心血管不良事件 OR 心脏毒性 OR 心血管毒性

Note: ROC:receiver operating curves; PD-1: Programmed cell death protein 1; PD-L1: Programmed death-ligand 1; CTLA-4: cytotoxic t-lymphocyte-associated antigen-4; ICIs: immune checkpoint inhibitors; irAEs: immune-related adverse events.

Chinese Biomedical literature Database

 ( "预测模型"[常用字段:智能] OR "风险模型"[常用字段:智能] OR "预测工具"[常用字段:智能] OR "风险评分"[常用字段:智能] OR "风险评估"[常用字段:智能] OR "预测因子"[常用字段:智能] OR "生物标志物"[常用字段:智能] OR "模型"[常用字段:智能] OR "人工智能"[常用字段:智能] OR "机器学习"[常用字段:智能] OR "深度学习"[常用字段:智能] OR "列线图"[常用字段:智能] OR "诺莫图"[常用字段:智能] OR "区分度"[常用字段:智能] OR "校准度"[常用字段:智能] OR "ROC曲线"[常用字段:智能] OR "决策曲线"[常用字段:智能]) AND( "免疫治疗"[常用字段:智能] OR "免疫检查点抑制剂"[常用字段:智能] OR "程序性死亡受体-1"[常用字段:智能] OR "程序性死亡配体-1"[常用字段:智能] OR "细胞毒性T淋巴细胞相关抗原-4"[常用字段:智能] OR "PD-1"[常用字段:智能] OR "PD-L1"[常用字段:智能] OR "CTLA-4"[常用字段:智能] OR "ICIs"[常用字段:智能]) AND( "免疫相关不良事件"[常用字段:智能] OR "不良反应"[常用字段:智能] OR "毒性"[常用字段:智能] OR "副作用"[常用字段:智能] OR "irAEs"[常用字段:智能] OR "症状"[常用字段:智能] OR "免疫治疗相关心脏毒性"[常用字段:智能] OR "免疫治疗相关心血管毒性"[常用字段:智能] OR "免疫治疗相关心肌炎"[常用字段:智能] OR "免疫检查点抑制剂相关心肌炎"[常用字段:智能] OR "免疫检查点抑制剂相关心脏毒性"[常用字段:智能] OR "免疫检查点抑制剂相关心血管毒性"[常用字段:智能] OR "静脉血栓"[常用字段:智能] OR "心律失常"[常用字段:智能] OR "心脏功能障碍"[常用字段:智能] OR "动脉粥样硬化"[常用字段:智能] OR "急性冠状动脉综合征"[常用字段:智能] OR "心包炎"[常用字段:智能] OR "血管炎"[常用字段:智能] OR "心肌炎"[常用字段:智能] OR "心脏不良事件"[常用字段:智能] OR "心血管不良事件"[常用字段:智能] OR "心脏毒性"[常用字段:智能] OR "心血管毒性"[常用字段:智能])

Note: ROC:receiver operating curves; PD-1: Programmed cell death protein 1; PD-L1: Programmed death-ligand 1; CTLA-4: cytotoxic t-lymphocyte-associated antigen-4; ICIs: immune checkpoint inhibitors; irAEs: immune-related adverse events.

Chinese Medical Association Full-Text Journal Database

(((*预测模型 OR 风险模型 OR 预测工具 OR 风险评分 OR 风险评估 OR 预测因子 OR 生物标志物 OR 模型 OR 人工智能 OR 机器学习 OR 深度学习 OR 列线图 OR 诺莫图 OR 区分度 OR 校准度 OR ROC曲线 OR 决策曲线*)) AND (免疫治疗 OR 免疫检查点抑制剂 OR 程序性死亡受体-1 OR 程序性死亡配体-1 OR 细胞毒性T淋巴细胞相关抗原-4 OR PD-1 OR PD-L1 OR CTLA-4 OR ICIs)) AND (免疫相关不良事件 OR 不良反应 OR 毒性 OR 副作用 OR irAEs OR 症状 OR 免疫治疗相关心脏毒性 OR 免疫治疗相关心血管毒性 OR 免疫治疗相关心肌炎 OR 免疫检查点抑制剂相关心肌炎 OR 免疫检查点抑制剂相关心脏毒性 OR 免疫检查点抑制剂相关心血管毒性 OR 静脉血栓 OR 心律失常 OR 心脏功能障碍 OR 动脉粥样硬化 OR 急性冠状动脉综合征 OR 心包炎 OR 血管炎 OR 心肌炎 OR 心脏不良事件 OR 心血管不良事件 OR 心脏毒性 OR 心血管毒性)

Note: PD-1: Programmed cell death protein 1; PD-L1: Programmed death-ligand 1; CTLA-4: cytotoxic t-lymphocyte-associated antigen-4; ICIs: immune checkpoint inhibitors; irAEs: immune-related adverse events.

Web of science

"prediction model" OR "forecast model" OR "predictive model" OR "prognostic models" OR "risk model" OR "predictive tool" OR "prediction tool" OR "risk score" OR "risk assessment" OR "risk prediction" OR "predictive factor" OR "biomarker" OR "model" OR "artificial intelligence" OR "machine learning" OR "deep learning" OR "nomogram" OR "discrimination" OR "calibration" OR "ROC curve" OR "C statistic" OR "area under curve" OR "decision curve analysis" (Topic) and "immune checkpoint inhibitors" OR "immunotherapy" OR "immune checkpoint blocker" OR "programmed death receptor" OR "programmed cell death protein" OR "programmed death ligand" OR "programmed cell death-ligand" OR "cytotoxic T-lymphocyte-associated antigen" OR "PD 1" OR "PD L1" OR "CTLA 4" OR "ICI*" (Topic) and "drug-related side effects and adverse reactions" OR "immune-related adverse reaction" OR "immune-related adverse events" OR "adverse reactions" OR "toxicity" OR "side effects" OR "irAEs" OR "symptoms" OR "immunotherapy-related cardiotoxicity" OR "immunotherapy-related cardiovascular toxicity" OR "immunotherapy-related myocarditis" OR "immune checkpoint inhibitor-related myocarditis" OR "immune checkpoint inhibitor-related cardiotoxicity" OR "immune checkpoint inhibitor-related cardiovascular toxicity" OR "immunotherapy-associated cardiotoxicity" OR "immunotherapy-associated cardiovascular toxicity" OR "immunotherapy-associated myocarditis" OR "immune checkpoint inhibitor-associated myocarditis" OR "immune checkpoint inhibitor-associated cardiotoxicity" OR "immune checkpoint inhibitor-associated cardiovascular toxicity" OR "Venous thromboembolism" OR "Arrhythmia" OR "Cardiac dysfunction" OR "Atherosclerosis" OR "Acute coronary syndrome" OR "Pericarditis" OR "Vasculitis" OR "cardiac adverse events" OR "cardiovascular adverse events" OR "cardiac toxicity" OR "cardiovascular toxicity" OR "myocarditis" (Topic)

Note: ROC:receiver operating curves; PD 1: Programmed cell death protein 1; PD L1: Programmed death-ligand 1; CTLA 4: cytotoxic t-lymphocyte-associated antigen-4; ICI: immune checkpoint inhibitor; irAEs: immune-related adverse events.

Pubmed

((("prediction model"[Title/Abstract] OR "forecast model"[Title/Abstract] OR "predictive model"[Title/Abstract] OR "prognostic models"[Title/Abstract] OR "risk model"[Title/Abstract] OR "predictive tool"[Title/Abstract] OR "prediction tool"[Title/Abstract] OR "risk score"[Title/Abstract] OR "risk assessment"[Title/Abstract] OR "risk prediction"[Title/Abstract] OR "predictive factor"[Title/Abstract] OR "biomarker"[Title/Abstract] OR "model"[Title/Abstract] OR "artificial intelligence"[Title/Abstract] OR "machine learning"[Title/Abstract] OR "deep learning"[Title/Abstract] OR "nomogram"[Title/Abstract] OR "discrimination"[Title/Abstract] OR "calibration"[Title/Abstract] OR "ROC curve"[Title/Abstract] OR "C statistic"[Title/Abstract] OR "area under curve"[Title/Abstract] OR "decision curve analysis"[Title/Abstract])) AND ((Immune Checkpoint Inhibitors[Mesh]) OR (("immunotherapy"[Title/Abstract] OR "immune checkpoint blocker"[Title/Abstract] OR "programmed death receptor"[Title/Abstract] OR "programmed cell death protein"[Title/Abstract] OR "programmed death ligand"[Title/Abstract] OR "programmed cell death-ligand"[Title/Abstract] OR "cytotoxic T-lymphocyte-associated antigen"[Title/Abstract] OR "PD 1"[Title/Abstract] OR "PD L1"[Title/Abstract] OR "CTLA 4"[Title/Abstract] OR "ICI*"[Title/Abstract])))) AND ((Drug-Related Side Effects and Adverse Reactions[Mesh]) OR (("immune-related adverse reaction"[Title/Abstract] OR "immune-related adverse events"[Title/Abstract] OR "adverse reactions"[Title/Abstract] OR "toxicity"[Title/Abstract] OR "side effects"[Title/Abstract] OR "irAEs"[Title/Abstract] OR "symptoms"[Title/Abstract] OR "immunotherapy-related cardiotoxicity"[Title/Abstract] OR "immunotherapy-related cardiovascular toxicity"[Title/Abstract] OR "immunotherapy-related myocarditis"[Title/Abstract] OR "immune checkpoint inhibitor-related myocarditis"[Title/Abstract] OR "immune checkpoint inhibitor-related cardiotoxicity"[Title/Abstract] OR "immune checkpoint inhibitor-related cardiovascular toxicity"[Title/Abstract] OR "immunotherapy-associated cardiotoxicity"[Title/Abstract] OR "immunotherapy-associated cardiovascular toxicity"[Title/Abstract] OR "immunotherapy-associated myocarditis"[Title/Abstract] OR "immune checkpoint inhibitor-associated myocarditis"[Title/Abstract] OR "immune checkpoint inhibitor-associated cardiotoxicity"[Title/Abstract] OR "immune checkpoint inhibitor-associated cardiovascular toxicity"[Title/Abstract] OR "Venous thromboembolism"[Title/Abstract] OR "Arrhythmia"[Title/Abstract] OR "Cardiac dysfunction"[Title/Abstract] OR "Atherosclerosis"[Title/Abstract] OR "Acute coronary syndrome"[Title/Abstract] OR "Pericarditis"[Title/Abstract] OR "Vasculitis"[Title/Abstract] OR "cardiac adverse events"[Title/Abstract] OR "cardiovascular adverse events"[Title/Abstract] OR "cardiac toxicity"[Title/Abstract] OR "cardiovascular toxicity"[Title/Abstract] OR "myocarditis"[Title/Abstract])))

Note: ROC:receiver operating curves; PD 1: Programmed cell death protein 1; PD L1: Programmed death-ligand 1; CTLA 4: cytotoxic t-lymphocyte-associated antigen-4; ICI: immune checkpoint inhibitor; irAEs: immune-related adverse events.

Embase

#1：'predictive model'/exp

#2：'prediction model':ab,ti OR 'forecast model':ab,ti OR 'predictive model':ab,ti OR 'prognostic models':ab,ti OR 'risk model':ab,ti OR 'predictive tool':ab,ti OR 'prediction tool':ab,ti OR 'risk score':ab,ti OR 'risk assessment':ab,ti OR 'risk prediction':ab,ti OR 'predictive factor':ab,ti OR 'biomarker':ab,ti OR 'model':ab,ti OR 'artificial intelligence':ab,ti OR 'machine learning':ab,ti OR 'deep learning':ab,ti OR 'nomogram':ab,ti OR 'discrimination':ab,ti OR 'calibration':ab,ti OR 'roc curve':ab,ti OR 'c statistic':ab,ti OR 'area under curve':ab,ti OR 'decision curve analysis':ab,ti

#3：'immune checkpoint inhibitor'/exp

#4：'immune checkpoint inhibitors':ab,ti OR 'immunotherapy':ab,ti OR 'immune checkpoint blocker':ab,ti OR 'programmed death receptor':ab,ti OR 'programmed cell death protein':ab,ti OR 'programmed death ligand':ab,ti OR 'programmed cell death-ligand':ab,ti OR 'cytotoxic t-lymphocyte-associated antigen':ab,ti OR 'pd 1':ab,ti OR 'pd l1':ab,ti OR 'ctla 4':ab,ti OR 'ici*':ab,ti

#5：'adverse drug reaction'/exp

#6：'drug-related side effects':ab,ti AND 'adverse reactions':ab,ti OR 'immune-related adverse reaction':ab,ti OR 'immune-related adverse events':ab,ti OR 'adverse reactions':ab,ti OR 'toxicity':ab,ti OR 'side effects':ab,ti OR 'iraes':ab,ti OR 'symptoms':ab,ti OR 'immunotherapy-related cardiotoxicity':ab,ti OR 'immunotherapy-related cardiovascular toxicity':ab,ti OR 'immunotherapy-related myocarditis':ab,ti OR 'immune checkpoint inhibitor-related myocarditis':ab,ti OR 'immune checkpoint inhibitor-related cardiotoxicity':ab,ti OR 'immune checkpoint inhibitor-related cardiovascular toxicity':ab,ti OR 'immunotherapy-associated cardiotoxicity':ab,ti OR 'immunotherapy-associated cardiovascular toxicity':ab,ti OR 'immunotherapy-associated myocarditis':ab,ti OR 'immune checkpoint inhibitor-associated myocarditis':ab,ti OR 'immune checkpoint inhibitor-associated cardiotoxicity':ab,ti OR 'immune checkpoint inhibitor-associated cardiovascular toxicity':ab,ti OR 'venous thromboembolism':ab,ti OR 'arrhythmia':ab,ti OR 'cardiac dysfunction':ab,ti OR 'atherosclerosis':ab,ti OR 'acute coronary syndrome':ab,ti OR 'pericarditis':ab,ti OR 'vasculitis':ab,ti OR 'cardiac adverse events':ab,ti OR 'cardiovascular adverse events':ab,ti OR 'cardiac toxicity':ab,ti OR 'cardiovascular toxicity':ab,ti OR 'myocarditis':ab,ti

#7：#1 OR #2

#8：#3 OR #4

#9：#5 OR #6

#10：#7 AND #8 AND #9

Note: roc:receiver operating curves; pd 1: Programmed cell death protein 1; pd l1: Programmed death-ligand 1; ctla 4: cytotoxic t-lymphocyte-associated antigen-4; ici: immune checkpoint inhibitor; iraes: immune-related adverse events.

Cochrane

"prediction model" OR "forecast model" OR "predictive model" OR "prognostic models" OR "risk model" OR "predictive tool" OR "prediction tool" OR "risk score" OR "risk assessment" OR "risk prediction" OR "predictive factor" OR "biomarker" OR "model" OR "artificial intelligence" OR "machine learning" OR "deep learning" OR "nomogram" OR "discrimination" OR "calibration" OR "ROC curve" OR "C statistic" OR "area under curve" OR "decision curve analysis" in Title Abstract Keyword AND "immune checkpoint inhibitors" OR "immunotherapy" OR "immune checkpoint blocker" OR "programmed death receptor" OR "programmed cell death protein" OR "programmed death ligand" OR "programmed cell death-ligand" OR "cytotoxic T-lymphocyte-associated antigen" OR "PD 1" OR "PD L1" OR "CTLA 4" OR "ICI*" in Title Abstract Keyword AND "drug-related side effects and adverse reactions" OR "immune-related adverse reaction" OR "immune-related adverse events" OR "adverse reactions" OR "toxicity" OR "side effects" OR "irAEs" OR "symptoms" OR "immunotherapy-related cardiotoxicity" OR "immunotherapy-related cardiovascular toxicity" OR "immunotherapy-related myocarditis" OR "immune checkpoint inhibitor-related myocarditis" OR "immune checkpoint inhibitor-related cardiotoxicity" OR "immune checkpoint inhibitor-related cardiovascular toxicity" OR "immunotherapy-associated cardiotoxicity" OR "immunotherapy-associated cardiovascular toxicity" OR "immunotherapy-associated myocarditis" OR "immune checkpoint inhibitor-associated myocarditis" OR "immune checkpoint inhibitor-associated cardiotoxicity" OR "immune checkpoint inhibitor-associated cardiovascular toxicity" OR "Venous thromboembolism" OR "Arrhythmia" OR "Cardiac dysfunction" OR "Atherosclerosis" OR "Acute coronary syndrome" OR "Pericarditis" OR "Vasculitis" OR "cardiac adverse events" OR "cardiovascular adverse events" OR "cardiac toxicity" OR "cardiovascular toxicity" OR "myocarditis" in Title Abstract Keyword - (Word variations have been searched)

Note: ROC:receiver operating curves; PD 1: Programmed cell death protein 1; PD L1: Programmed death-ligand 1; CTLA 4: cytotoxic t-lymphocyte-associated antigen-4; ICI: immune checkpoint inhibitor; irAEs: immune-related adverse events.

Table 1-1 Candidate predictors and outcome diagnostic criteria/definitions of prediction model for the occurrence risk of overall immune checkpoint inhibitor-related cardiovascular toxicity

| No | Author (year) | Model number | Candidate variables | Prediction outcome diagnostic criteria/definitions |
| --- | --- | --- | --- | --- |
| 1 | Zhihui Yan 2026 | Model 1 | Gender; Eastern Cooperative Oncology Group performance status; Smoking; Drinking; Tumor stage; Combined hypertension; Diabetes mellitus; Coronary heart disease; Combined therapy; Tumor metastasis; Age; Systemic inflammation response index; Body mass index. | Cardiovascular toxicity outcomes included myocarditis, non malignant pericardial disease, acute coronary syndrome, congestive heart failure, atrioventricular block, supraventricular and ventricular arrhythmias, stress cardiomyopathy (Takotsubo syndrome), vasculitis, and venous thromboembolism. The composite endpoint “cardiovascular toxicity” included all cardiovascular events occurring during ICI treatment, irrespective of whether they were adjudicated as immunemediated. Definitions for each event were based on the 2022 European Society of Cardiology Cardio-Oncology Guidelines. |
| 2 | Chadi Ayoub 2025 | Model 1-3 | Not clearly reported | Outcomes included ICI-related myocarditis and major adverse cardiovascular events (cardiac mortality, new heart failure, transient ischemic attack /stroke, and myocardial infarction). ICI  related myocarditis was deﬁned based on the Bonaca et al diagnostic criteria. |
| 3 | Jialian Li 2025 | Model 1-3 | Age; Gender; Smoke; Alcohol; Systolic blood pressure; Diastolic blood pressure; Body mass index; Hypertension; Diabetes mellitus; Coronary artery disease; ICI strategy; Cardiac troponin T; Creatine kinase-MB; Myoglobin; N-terminal pro B-type Natriuretic Peptide; High-sensitivity C-reactive protein; C-reactive protein; Neutrophil-to-lymphocyte ratio; Eosinophil count; Eosinophil percentage; Basophil percentage; Creatinine; Total cholesterol; Low-density lipoprotein; Potassium; Interleukin-6; Interleukin-17A; T wave; R wave amplitude in V5 lead; S wave amplitude in V1 lead; QT interval; Corrected QT interval using Fridericia’s formula; QRS duration; PR interval; Sokolow-Lyon index. | According to the 2020 National Comprehensive Cancer Network Guidelines and the 2018 American Society of Clinical Oncology Guidelines, to predict immunotherapy-related cardiotoxicity early, the observed outcome is defined as abnormalities in cardiac biomarkers, particularly cardiac troponin and electrocardiogram abnormalities. Abnormal cardiac biomarkers are defined as values exceeding the upper limit of normal (ULN), with a further increase if the baseline is already above the ULN. Abnormal electrocardiogram is defined according to the 2022 European Society of Cardiology Guidelines. |

Table 1-1 Candidate predictors and outcome diagnostic criteria/definitions of prediction model for the occurrence risk of overall immune checkpoint inhibitor-related cardiovascular toxicity (continued)

| No | Author (year) | Model number | Candidate variables | Prediction outcome diagnostic criteria/definitions |
| --- | --- | --- | --- | --- |
| 4 | Qiuyue Li 2024 | Model 1 | Gender；Age；Body mass index；Tumor stage；Pathological type；History of lobectomy；Hypertension；Diabetes mellitus；Hyperlipidemia；Smoking；Drinking；Coronary heart disease；Medication regimen；Combined targeted therapy；Complicated with other immune-related adverse events；N-terminal pro-brain natriuretic peptide；Total cholesterol；Triglyceride；High-density lipoprotein；Low-density lipoprotein；Neutrophil-to-Lymphocyte Ratio；Creatine kinase；Creatine kinase isoenzyme MB；α-Hydroxybutyrate Dehydrogenase；Aspartate aminotransferase；Lactate dehydrogenase；Myoglobin. | The diagnostic criteria for immunotherapy-related cardiotoxicity were mainly based on the 2023 Chinese Society of Clinical Oncology Guidelines for Cardio-Oncology and the 2022 European Society of Cardiology Cardio-Oncology Guidelines. Diagnosis was comprehensively determined by integrating abnormal findings on electrocardiography, troponin levels, and transthoracic echocardiography together with clinical manifestations. The presence of any single abnormal parameter was sufficient to confirm the occurrence of immunotherapy-related cardiotoxicity. |
| 5 | Xitong Cheng  2024 | Model 1-Model 44 | Age；Gender；Height；Weight；Body mass index；History of drinking；History of smoking；Hypertension；Diabetes；Autoimmune disease；Cancer type；Tumor node metastasis classification；Surgical history；History of radiotherapy；Anti-programmed cell death protein 1/programmed death-ligand 1 types；Course of treatment；Chemotherapeutics；Targeted therapeutic drugs；Antibiotics；Oralg lucocorticoids；Inhaled/topical steroids；Nonsteroidal anti-inflammatory drugs；Insulin；Angiotension converting enzyme inhibitors/Angiotonin receptor blocker；Morphine；Immunomodulators；Calcium channel blockers；Psychotropic drugs；Beta-blocker；Diurtic；N-terminal B-type natriuretic peptide；Creatine kinase MB；Cardiac troponin T；Left ventricular ejection fraction；C-reactive protein；Interleukin-2；Interleukin-4；Interleukin-6；Interleukin-10；Cluster of Differentiation 3；Cluster of Differentiation 4；Cluster of Differentiation 8；Interferon-γ；Natural killer cell；Tumor Necrosis Factor；Albumin；Leukocyte；Monocyte；Lymphocyte；Eosinophils；Neutrophils. | The immune-related cardiac adverse events were diagnosed and graded according to the 2022 European Society of Cardiology Guidelines on cardio-oncology and Common Terminology Criteria for Adverse Events (CTCAE,Version 5.0).  . |

Table 1-1 Candidate predictors and outcome diagnostic criteria/definitions of prediction model for the occurrence risk of overall immune checkpoint inhibitor-related cardiovascular toxicity (continued)

| No | Author (year) | Model number | Candidate variables | Prediction outcome diagnostic criteria/definitions |
| --- | --- | --- | --- | --- |
| 6 | Samuel Peter Heilbroner  2021 | Model 1 | Not clearly reported | A cardiac event was defined as the first documentation of arrhythmia (eg, complete heart block and ventricular fibrillation), heart failure, myocarditis, or pericardial disease as defined by the Medical Dictionary for Regulatory Activities (MedDRA) and International Classification of Diseases (ICD) codes. |

Table 1-2 Candidate predictors and outcome diagnostic criteria/definitions of prediction model for the occurrence risk of immune checkpoint inhibitor-related myocarditis

| No | Author (year) | Model number | Candidate variables | Prediction outcome diagnostic criteria/definitions |
| --- | --- | --- | --- | --- |
|  |  |  |  |  |
| 1 | Shaqi Rao 2026 | Model 1 | Gender；Age；Body mass index；Tumor type（Lung cancer、Liver cancer、Gastric cancer、Other malignancies）；Prior medical history（None、Hypertension、Diabetes mellitus、Other comorbidities）；Prior anti-tumor therapy（None、Chemotherapy、Surgery、Radiotherapy、Targeted therapy）；Type of immune checkpoint inhibitors（Programmed cell death protein 1 inhibitor、Programmed death-ligand 1 inhibitor）；Immune checkpoint inhibitors combined with chemotherapy；Post-treatment Creatine Kinase；Percent change in Creatine Kinase；Post-treatment Creatine Kinase-MB isoenzyme；Percent change in Creatine Kinase-MB isoenzyme；Post-treatment Lactate Dehydrogenase；Percent change in Lactate Dehydrogenase；Post-treatment Alanine Aminotransferase；Percent change in Alanine Aminotransferase；Post-treatment Aspartate Aminotransferase；Percent change in Aspartate Aminotransferase；Post-treatment Neutrophil percentage；Percent change in Neutrophil percentage；Post-treatment Lymphocyte percentage；Percent change in Lymphocyte percentage；Post-treatment Monocyte percentage；Percent change in Monocyte percentage；Post-treatment Heart Rate；Percent change in Heart Rate；Post-treatment P-wave duration；Percent change in P-wave duration；Post-treatment PR interval；Percent change in PR interval；Post-treatment QRS duration；Percent change in QRS duration；Post-treatment QRS axis；Percent change in QRS axis；Post-treatment Sokolow-Lyon index；Percent change in Sokolow-Lyon index；Post-treatment corrected QT interval；Percent change in corrected QT interval；T wave morphology（Normal、Inverted、Biphasic、Peaked、Flat）. | The assessment of myocarditis was based on the position statement of the European Society of Cardiology |

Table 1-2 Candidate predictors and outcome diagnostic criteria/definitions of prediction model for the occurrence risk of immune checkpoint inhibitor-related myocarditis (continued)

| No | Author (year) | Model number | Candidate variables | Prediction outcome diagnostic criteria/definitions |
| --- | --- | --- | --- | --- |
| 2 | Tao Luan  2026 | Model 1 | Age；Gender；Smoking；Comorbidity；Lung cancer type；Clinical stage；First/Non-first treatment；Heart failure；Troponin；Creatine kinase；B-type natriuretic peptide；Electrocardiogram；Echocardiography；Interleukin-1β；Interleukin-6；Interleukin-8. | Immune checkpoint inhibitors-associated myocarditis was adjudicated by two experienced physicians. After excluding alternative etiologies, diagnosis integrated medical history and clinical symptoms, the temporal relationship between immune checkpoint inhibitors exposure and symptom onset, dynamic changes in troponin and other myocardial injury biomarkers,imaging abnormalities, cardiac magnetic resonance (CMR), and endomyocardial biopsy when indicated. Toxicity grading followed Common Terminology Criteria for Adverse Events Version 5.0,with immune checkpoint inhibitors-associated myocarditis classified as mild (grades 1-2) or severe (grade≥3). |
| 3 | Zhuoling Zheng  2026 | Model 1 | Not clearly reported | The diagnosis of myocarditis: according to the 2025 European Society of Cardiology Guidelines for the management of myocarditis and pericarditis. |
| 4 | Reina Yamamoto 2026 | Model 1 | Quantitative variables（Age; Body mass index; Barthel index; Charlson index; Prescription count） Qualitative variables（585，not clearly reported）. | International Classification of Diseases, 10th Revision code： I401 Isolated myocarditis； I408 Other acute myocarditis； I409 Acute myocarditis, unspecified； I514 Myocarditis, unspecified. |
| 5 | Shanshan Li (1) 2025 | Model 1 | Age；Gender；Pathological type；Staging；The number of times ICIs；α-hydroxybutyrate dehydrogenase；Creatine kinase-M/B；High-sensitivity cardiac troponin T；The multiple of N-terminal pro-brain natriuretic peptide；Coronary artery disease；Atrial fibrillation；Hypertension；Diabetes；Cerebral infarction；Concomitant medications；Radiotherapy；Caprini risk score. | The assessment of myocarditis was based on the 2022 European Society of Cardiology Cardio-Oncology Guidelines. |

Table 1-2 Candidate predictors and outcome diagnostic criteria/definitions of prediction model for the occurrence risk of immune checkpoint inhibitor-related myocarditis (continued)

| No | Author (year) | Model number | Candidate variables | Prediction outcome diagnostic criteria/definitions |
| --- | --- | --- | --- | --- |
| 6 | Shanshan Li (2) 2025 | Model 1-5 | Age；Gender；Histological type；Tumor stage；α-hydroxybutyrate dehydrogenase；Creatine kinase MB；High-sensitivity cardiac troponin T；N-terminal pro-B-type natriuretic peptide；Immune checkpoint inhibitorss treatment cycle；Electrocardiogram-score；Coronary heart disease；Hypertension；Diabetes mellitus；Cerebral infarction；Left thoracic/mediastinal radiotherapy；Medication therapy. | The assessment of myocarditis was based on the 2022 European Society of Cardiology Cardio-Oncology Guidelines. |

Table 1-3 Candidate predictors and outcome diagnostic criteria/definitions of prediction model for the occurrence risk of immune checkpoint inhibitor-related venous thromboembolism

| No | Author (year) | Model number | Candidate variables | Prediction outcome diagnostic criteria/definitions |
| --- | --- | --- | --- | --- |
| 1 | Yunhan Su 2026 | Model 1 | Age；Gender；Body mass index；Smoking；Squamous cell carcinoma；Adenocarcinoma；Small cell lung cancer；Other histological types；TNM stage；Eastern Cooperative Oncology Group performance status；Programmed cell death ligand 1 expression；Hypertension；Diabetes mellitu；Hyperlipidemia；Severe pulmonary disease；Respiratory failure；Atrial fibrillation；Lower extremity edema；Abnormal pulmonary ventilation function；History of venous thromboembolism；Hemoglobin；Hemoglobin < 10 g/dL；White blood cell；White blood cell > 11×10⁹/L；Platelet；Platelet ≥ 350×10⁹/L；D-dimer；Chemotherapy；Thoracic radiotherapy；Targeted therapy；Surgical treatment；Line of immune checkpoint inhibitor therapy；Treatment regimen；Central venous catheterization；Glucocorticoid therapy. | Venous thromboembolism events included deep vein thrombosis and pulmonary embolism. All cases were confirmed by imaging examinations. |
| 2 | Xiaotong Xia 2025 | Model 1 | Not clearly reported | The venous thromboembolism was defined as a composite of deep venous thrombosis or pulmonary embolism confirmed by standardized radiographic findings. |

Table 1-3 Candidate predictors and outcome diagnostic criteria/definitions of prediction model for the occurrence risk of immune checkpoint inhibitor-related venous thromboembolism (continued)

| No | Author (year) | Model number | Candidate variables | Prediction outcome diagnostic criteria/definitions |
| --- | --- | --- | --- | --- |
| 3 | Yihuai Hu 2024 | Model 1 | Age；Gender；Smoking；Drinking；Hypertension；Diabetes mellitus；History of heart disease；Eastern Cooperative Oncology Group performance status；Body mass index；Tumor location；Tumor subtype；Programmed death-ligand 1 expression level；T stage；N stage；M stage；Clinical stage；Surgical history；Chemotherapeutic drugs；Targeted drugs；Anti-angiogenic drugs；Radiotherapy；White blood cell；Hemoglobin；Platelet；Neutrophil；Lymphocyte；Squamous cell carcinoma antigen；Cytokeratin 19 fragment；Carcinoembryonic antigen；Neuron-specific enolase；Pro-gastrin-releasing peptide；Prothrombin time-international normalized ratio；Antithrombin Ⅲ activity；Thrombin time；D-dimer；Prothrombin time；Activated partial thromboplastin time；Fibrinogen；Blood urea nitrogen；γ-glutamyl transferase；Uric acid；Alkaline phosphatase；Alanine aminotransferase；Triglyceride；Creatinine；Aspartate aminotransferase；Lactate dehydrogenase；Globulin；Albumin；Total protein；High-sensitivity C-reactive protein；Tumor specific growth factor；Myoglobin；Cardiac Troponin T；N-terminal pro-B-type Natriuretic Peptide. | Definition of immune checkpoint inhibitor therapy-associated venous thromboembolism: Deep vein thrombosis or pulmonary embolism diagnosed by imaging examination after the initial administration of immune checkpoint inhibitors. |
| 4 | Guanzhong Liang 2024 | Model 1 | Age；Gender；Body mass index；Karnofsky Pperformance status score；TNM staging；Surgical history；Radiotherapy history；Chemotherapy history；Targeted therapy；Platelet count；Lactate dehydrogenase；Monoamine oxidase；Prothrombin time；Activated partial thromboplastin time；D-dimer；Fibrinogen；Fibrin degradation products；T cell；B cell；NK cell ratios；White blood cell；Hemoglobin. | All venous thromboembolism patients are diagnosed by clinical specialist physicians in conjunction with ultrasound or imaging examinations. |
| 5 | Jiarui Zhang 2024 | Model 1-3 | Not applicable (not for model development) | Venous thromboembolism was defined as symptomatic or asymptomatic pulmonary embolism or deep venous thrombosis based on standardized radiographic findings. |

Table 1-4 Candidate predictors and outcome diagnostic criteria/definitions of prediction model for the risk of mortality following immune checkpoint inhibitor-related cardiovascular toxicity

| No | Author (year) | Model number | Candidate variables | Prediction outcome diagnostic criteria/definitions |
| --- | --- | --- | --- | --- |
| 1 | Zhihui Yan 2025 | Model 1 | Gender；Eastern Cooperative Oncology Group performance status；Smoking；Drinking；Tumor stage；Hypertension；Diabetes mellitus；Coronary heart disease；Types of immune checkpoint inhibitors；Combined therapy；Other immune-related adverse events；Tumor metastasis；Echocardiographic ventricular wall motion abnormalities；Abnormal ST-T segments；Second to third degree atrioventricular block；Age；Body bass index；White blood cell；Neutrophil；Lymphocyte；Platelet；Systemic inflammatory response index；Systemic immune-inflammation index；Advanced systemic inflammatory index；Monocyte. | Diagnostic criteria for immune checkpoint inhibitor treatment-related cardiovascular toxicity: 2022 European Society of Cardiology Cardio-Oncology Guidelines, 2023 Chinese Society of Clinical Oncology Guidelines for Management of Immune checkpoint inhibitor-Related Toxicities, and Recommendations for Prevention, Treatment and Management of Immune checkpoint inhibitor-Related Toxicities. |
| 2 | Xiaoyun Cheng 2025 | Model 1 | Male；Age；Body mass index；Hypertension；Coronary artery disease；Immune-related adverse events；Smoke；Alcohol；Small cell lung cancer；Multiple metastas；Programmed death-ligand 1 expression in the tumor proportion score；Driver mutations；Chemotherapy；Programmed death-ligand 1；Antivascular；Radiotherapy；Targeting；Troponin；N-terminal pro brain natriuretic peptide；Severe myocarditis；Ejection fraction；Major adverse cardiovascular events；Severe myocarditis,High-dose glucocorticoids；Severe myocarditis, Low-dose glucocorticoids；Heart failure II；Heart failure III；Heart failure IV；Immune checkpoint inhibitors Duration≥132 days. | The definition of myocarditis followed the consensus statement from the International Cardio-Oncology Society and patients were categorized into possible/ probable/confirmed myocarditis according to the case definitions proposed by Bonaca et al. |
| 3 | Zhengkun Guan 2025 | Model 1-2 | Aspartate transferase-to-albumin ratio；Albumin；Alanine aminotransferase；Aspartate aminotransferase；Creatine kinase isoenzyme MB；Cardiac troponin-I；Absolute eosinophil count；Lactate dehydrogenase；Lactic dehydrogenase-to-albumin ratio；Absolute lymphocyte count；Monocyte-to-lymphocyte ratio；Absolute monocyte count；Absolute neutrophil count；Neutrophil-to-eosinophil ratio；Neutrophil-to-lymphocyte ratio；N-terminal pro-brain natriuretic peptide；Platelet-to-lymphocyte ratio；Platelet count；Systemic immune-inflammation index；System inflammation response index. | The diagnosis of immune checkpoint inhibitor myocarditis: according to 2022 European Society of Cardiology Cardio-Oncology Guidelines. Overall survival time was defined as the duration from the initial diagnosis of myocarditis to the occurrence of all-cause mortality. |

Table 1-5 Candidate predictors and outcome diagnostic criteria/definitions of prediction model for the risk of major adverse cardiovascular/cardiac events following immune checkpoint inhibitor-related cardiovascular toxicity

| No | Author (year) | Model number | Candidate variables | Prediction outcome diagnostic criteria/definitions |
| --- | --- | --- | --- | --- |
|  |  |  |  |  |
| 1 | John R. Power 2025 | Model 1 | Age；Body bass index；Female；Presence of thymoma; Concurrent vascular endothelial growth factor inhibitor；Immune checkpoint inhibitors Combination therapy；Time from current line 1st immune checkpoint inhibitor infusion to myocarditis；Cardiomuscular svmptoms；Myositis；Pathological Q waves；Sokolow–Lyon QRS voltage；QRS duration；Left ventricular ejection fraction；Creatine kinase；Troponin (preferentially T, otherwise I)；Neutrophil-to-lymphocyte ratio；Corticosteroids；Immunomodulators. | The diagnosis of immune checkpoint inhibitor myocarditis: according to Bonaca’s diagnostic criteria（training set）；the updated Bonaca’s criteria（test set）. Major cardiomyotoxic events was defined as any of the following: (i) severe heart failure requiring either intravenous medical therapy or mechanical circulatory support; (ii) severe arrhythmia defined as sustained ventricular arrhythmia, complete heart block, sudden cardiac death, use of atropine or isoproterenol, and/or use of pacemaker/defibrillator; (iii) respiratory failure due to myositis-related respiratory muscle failure requiring ventilation; and/or (iv) death due to these conditions (henceforth referred to as cardiomyotoxicities). |
| 2 | Zhenli Li 2024 | Model 2 | Lactate dehydrogenase-to-albumin ratio（LAR)；Aspartate aminotransferase-to-albumin ratio(AAR)；Platelet-to-lymphocyte ratio(PLR)；Neutrophil-to-lymphocyte ratio(NLR)；Neutrophil-to-eosinophil ratio(NER) and Neutrophil-to-high-density lipoprotein cholesterol ratio(NHR) at the onset of myocarditis. | Immune checkpoint inhibitors‑related myocarditis was diagnosed according to the guidelines of the European Society of Cardiology(2013 version) and the major adverse cardiovascular events were defined as cardiovascular death, cardiac arrest, cardiogenic shock, and hemodynamically significant complete heart block that happened after the diagnosis of immune checkpoint inhibitor-associated myocarditis for the first time. |
| 3 | Rui Lu 2024 | Model 1-5 | Not clearly reported | The diagnosis of immune checkpoint inhibitor-associated myocarditis was based on the Chinese expert consensus on the surveillance and management of immune checkpoint inhibitor-related myocarditis (2020 version). Major adverse cardiovascular events were defined as severe heart failure, malignant arrhythmia, cardiogenic shock, and death. |

Table 1-6 Candidate predictors and outcome diagnostic criteria/definitions of diagnostic prediction model for grading the severity of immune checkpoint inhibitor-related cardiovascular toxicity

| No | Author (year) | Model number | Candidate variables | Prediction outcome diagnostic criteria/definitions |
| --- | --- | --- | --- | --- |
| 1 | Zhuoling Zheng  2026 | Model 2 | Not clearly reported | The diagnosis of myocarditis: according to the 2025 European Society of Cardiology Guidelines for the management of myocarditis and pericarditis.The severity of immune checkpoint inhibitor-associated myocarditis: according to the Common Terminology Criteria for Adverse Events criteria version 5.0 and grades 1 and 2 were combined as mild myocarditis, and grades 3 and 4 were combined as severe myocarditis. |
| 2 | Zhenli Li 2024 | Model 1 | Lactate dehydrogenase-to-albumin ratio（LAR)；Aspartate aminotransferase-to-albumin ratio(AAR)；Platelet-to-lymphocyte ratio(PLR)；Neutrophil-to-lymphocyte ratio(NLR)；Neutrophil-to-eosinophil ratio(NER) and Neutrophil-to-high-density lipoprotein cholesterol ratio(NHR) at the onset of myocarditis；the change rate of the above variables from baseline to the occurrence of myocarditis. | Immune checkpoint inhibitors‑related myocarditis was diagnosed according to the guidelines of the European Society of Cardiology (2013 version) and graded by the Common Terminology Criteria for Adverse Events criteria version 4.03. Grades ≤ 2 were classifed as mild myocarditis, whereas grades ≥ 3 were severe myocarditis. |

Table 2-1 Final predictors and frequency of prediction model for the occurrence risk of overall immune checkpoint inhibitor-related cardiovascular toxicity

| **No** | **Predictors** | **Frequency** |
| --- | --- | --- |
| 1 | N-terminal B-type natriuretic peptide | 8 |
| 2 | Age | 5 |
| 3 | Cardiac troponin T | 4 |
| 4 | C-reactive protein | 4 |
| 5 | Creatine kinase isoenzyme | 4 |
| 6 | Interleukin-2 | 4 |
| 7 | Diabetes | 3 |
| 8 | Hypertension | 3 |
| 9 | Corrected QT interval using Fridericia's formula | 2 |
| 10 | Gender | 2 |
| 11 | Interferon-γ | 2 |
| 12 | Left ventricular ejection fraction | 2 |
| 13 | QT interval | 2 |
| 14 | R wave amplitude in V5 lead | 2 |
| 15 | Systolic blood pressure | 2 |
| 16 | Atrial ﬁbrillation | 1 |
| 17 | Autoimmune disease | 1 |
| 18 | Basophil percentage | 1 |
| 19 | Body mass index | 1 |
| 20 | Calcium channel blockers | 1 |
| 21 | Cancer type | 1 |

Table 2-1 Final predictors and frequency of prediction model for the occurrence risk of overall immune checkpoint inhibitor-related cardiovascular toxicity (continued)

| **No** | **Predictors** | **Frequency** |
| --- | --- | --- |
| 22 | Chronic obstructive pulmonary disease | 1 |
| 23 | Combined targeted therapy | 1 |
| 24 | Coronary heart disease | 1 |
| 25 | Course of treatment | 1 |
| 26 | Creatinine | 1 |
| 27 | Eastern Cooperative Oncology Group performance status | 1 |
| 28 | Eosinophil count | 1 |
| 29 | Fam-history of coronary artery disease | 1 |
| 30 | History of radiotherapy | 1 |
| 31 | Hyperlipidemia | 1 |
| 32 | Immunomodulators | 1 |
| 33 | Interleukin-6 | 1 |
| 34 | Low density lipoprotein | 1 |
| 35 | Lung/chest cancer | 1 |
| 36 | Morphine | 1 |
| 37 | Myocardial infarction | 1 |
| 38 | Myoglobin | 1 |
| 39 | Obstructive sleep apnea | 1 |
| 40 | Oral glucocorticoids | 1 |
| 41 | Potassium | 1 |
| 42 | PR interval | 1 |

Table 2-1 Final predictors and frequency of prediction model for the occurrence risk of overall immune checkpoint inhibitor-related cardiovascular toxicity (continued)

| **No** | **Predictors** | **Frequency** |
| --- | --- | --- |
| 43 | Previous radiation | 1 |
| 44 | QRS duration | 1 |
| 45 | S wave amplitude in V1 lead | 1 |
| 46 | Smoking | 1 |
| 47 | Sokolow-Lyon index | 1 |
| 48 | Stroke/transient ischemic attack | 1 |
| 49 | Systemic inflammation response index | 1 |
| 50 | T wave | 1 |
| 51 | Tumor metastasis | 1 |
| 52 | Tumor stage | 1 |
| 53 | Ventricular tachycardia | 1 |
| 54 | α-hydroxybutyrate dehydrogenase | 1 |

Table 2-2 Final predictors and frequency of prediction model for the occurrence risk of immune checkpoint inhibitor-related myocarditis

| **No** | **Predictors** | **Frequency** |
| --- | --- | --- |
| 1 | B-type natriuretic peptide | 3 |
| 2 | High-sensitivity cardiac troponin T | 3 |
| 3 | Creatine kinase MB | 2 |
| 4 | α-hydroxybutyrate dehydrogenase | 2 |
| 5 | Age | 1 |
| 6 | Antipsychotics | 1 |
| 7 | Body mass index | 1 |
| 8 | Caprini risk score | 1 |
| 9 | Echocardiography | 1 |
| 10 | Electrocardiogram | 1 |
| 11 | Electrocardiogram-score | 1 |
| 12 | Elevated eosinophil ratio | 1 |
| 13 | Elevated myoglobin | 1 |
| 14 | Esophageal and gastric cancer | 1 |
| 15 | Hypertension | 1 |
| 16 | Immune checkpoint inhibitor combined use | 1 |
| 17 | Ipilimumab | 1 |
| 18 | Left thoracic/mediastinal radiotherapy | 1 |
| 19 | Lung cancer | 1 |
| 20 | Mucolytics | 1 |
| 21 | Osmotically acting laxatives | 1 |

Table 2-2 Final predictors and frequency of prediction model for the occurrence risk of immune checkpoint inhibitor-related myocarditis (continued)

| **No** | **Predictors** | **Frequency** |
| --- | --- | --- |
| 22 | Percent change in Standardized Sokolow-Lyon index | 1 |
| 23 | Prescription count | 1 |
| 24 | Prior existing disease | 1 |
| 25 | Prior tumor therapy | 1 |
| 26 | QRS Axis after immune checkpoint inhibitors thepy | 1 |
| 27 | Reduced lymphocyte ratio | 1 |
| 28 | Urologic cancer | 1 |

Table 2-3 Final predictors and frequency of prediction model for the occurrence risk of immune checkpoint inhibitor-related venous thromboembolism

| **No** | **Predictors** | **Frequency** |
| --- | --- | --- |
| 1 | Chemotherapy | 2 |
| 2 | D-dimer | 2 |
| 3 | Eastern Cooperative Oncology Group performance status | 2 |
| 4 | Smoke | 2 |
| 5 | Abnormal pulmonary ventilation function | 1 |
| 6 | Age | 1 |
| 7 | Body mass index | 1 |
| 8 | Diabetes mellitus | 1 |
| 9 | Fibrinogen | 1 |
| 10 | Glucocorticoid therapy | 1 |
| 11 | Heart disease | 1 |
| 12 | History of any cardiovascular event | 1 |
| 13 | History of venous thromboembolism | 1 |
| 14 | Khorana risk score ≥3 | 1 |
| 15 | Lactate dehydrogenase | 1 |
| 16 | Liver cancer | 1 |
| 17 | Liver dysfunction | 1 |
| 18 | Monoamine oxidase | 1 |
| 19 | Myoglobin | 1 |
| 20 | Platelet count | 1 |
| 21 | Respiratory failure | 1 |

Table 2-3 Final predictors and frequency of prediction model for the occurrence risk of immune checkpoint inhibitor-related venous thromboembolism (continued)

| **No** | **Predictors** | **Frequency** |
| --- | --- | --- |
| 22 | Severe pulmonary disease | 1 |
| 23 | Targeted therapy | 1 |
| 24 | White blood cell | 1 |

Table 2-4 Final predictors and frequency of prediction model for the risk of mortality following immune checkpoint inhibitor-related cardiovascular toxicity

| **No** | **Predictors** | **Frequency** |
| --- | --- | --- |
| 1 | Cardiac troponin-I | 2 |
| 2 | Lactic dehydrogenase-to-albumin ratio | 2 |
| 3 | N-terminal pro-brain natriuretic peptide | 2 |
| 4 | Combined radiotherapy | 1 |
| 5 | Diabetes | 1 |
| 6 | Duration of immune checkpoint inhibitor treatment | 1 |
| 7 | Echocardiographic ventricular wall motion abnormalities | 1 |
| 8 | Low-dose glucocorticoids in patients with severe myocarditis | 1 |
| 9 | Monocyte | 1 |
| 10 | System inflammation response index | 1 |
| 11 | Types of immune checkpoint inhibitors | 1 |

Table 2-5 Final predictors and frequency of prediction model for the risk of major adverse cardiovascular/cardiac events following immune checkpoint inhibitor-related cardiovascular toxicity

| **No** | **Predictors** | **Frequency** |
| --- | --- | --- |
| 1 | Left ventricular ejection fraction | 2 |
| 2 | Neutrophil to lymphocyte ratio | 2 |
| 3 | ST-T segment changes | 2 |
| 4 | Active thymoma | 1 |
| 5 | Elevated troponin | 1 |
| 6 | Lactate dehydrogenase-to-albumin ratio | 1 |
| 7 | Low Sokolow-Lyon QRS voltage on presenting electrocardiogram | 1 |
| 8 | Neutrophil-to-high-density lipoprotein cholesterol ratio | 1 |
| 9 | Presence of cardiomuscular symptoms | 1 |
| 10 | Second-to third-degree atrioventricular block | 1 |
| 11 | Ventricular tachycardia | 1 |
| 12 | White blood cell count | 1 |

Table 2-6 Final predictors and frequency of diagnostic prediction model for grading the severity of immune checkpoint inhibitor-related cardiovascular toxicity

| **No** | **Predictors** | **Frequency** |
| --- | --- | --- |
| 1 | Complete/incomplete bundle branch block | 1 |
| 2 | Creatine kinase elevation | 1 |
| 3 | Lactate dehydrogenase-to-albumin ratio at the onset of myocarditis | 1 |
| 4 | The change rate of Lactate dehydrogenase-to-albumin ratio from baseline to the occurrence of myocarditis | 1 |
| 5 | T-wave changes | 1 |

Table 3-1 **Characteristics** of prediction model for the occurrence risk of overall immune checkpoint inhibitor-related cardiovascular toxicity

| **No** | **Author (year)** | **Model number** | Prediction outcome | **Predictors  selection** | **Modeling methods** | **Candidate predictors** | | **Sample size (total/events number)** | | **Development cohort EPV** |
| --- | --- | --- | --- | --- | --- | --- | --- | --- | --- | --- |
|  |  |  |  |  |  | **Collection time** | **Continuous  variable  processing  method** | **Development cohort** | **External validation cohort** |  |
| 1 | Zhihui Yan 2026 | Model 1 | ICI treatment-related cardiovascular toxicity | Univariate/multivariate logistic regression analysis、according to literature and expert consultation | Logistic regression analysis | Not clearly reported | Continuous variables were transformed into categorical variables | 180/60 | None | 4.62 |
| 2 | Chadi Ayoub 2025 | Model 1-3 | Composite cardiovascular clinical adverse events including ICI-related myocarditis and major  adverse cardiovascular events | Not clearly reported | Model 1:Calibrated random forest  Model 2: One-Dimensional Convolutional Neural Network  Model 3: Joint fusion deep learning model | Not clearly reported | Continuous variables were transformed into categorical variables | 2258/264（Training set: 1807/not clearly reported；Test set: 451/not clearly reported） | None | Not clearly reported |

Table 3-1 **Characteristics** of prediction model for the occurrence risk of overall immune checkpoint inhibitor-related cardiovascular toxicity (continued)

| **No** | **Author (year)** | **Model number** | Prediction outcome | **Predictors  selection** | **Modeling methods** | **Candidate predictors** | | **Sample size (total/events number)** | | **Development cohort EPV** |
| --- | --- | --- | --- | --- | --- | --- | --- | --- | --- | --- |
|  |  |  |  |  |  | **Collection time** | **Continuous  variable  processing  method** | **Development cohort** | **External validation cohort** |  |
| 3 | Jialian Li 2025 | Model 1-3 | ICI-related cardiotoxicity | Model 1: Stepwise regression  Model 2: Feature importance analyse  Model 3: Backward stepwise selection | Model 1: Machine learning (Logistic Regression algorithm）  Model 2: Machine learning (Random Forest algorithm） Model 3: Machine learning (eXtreme Gradient Boosting (XGBoost) algorithm) | Before initiation of ICIs therapy | Retained as continuous variables | 419/78（Training set: 293/not clearly reported；Test set: 126/not clearly reported） | None | Not clearly reported |
| 4 | Qiuyue Li 2024 | Model 1 | Immunotherapy-related cardiotoxicity | Univariate analysis、 multivariate logistic regression analysis | Logistic regression analysis | The baseline data before receiving immunotherapy | Partial variables were transformed into categorical variables | 184/62 | None | 2.3 |

Table 3-1 **Characteristics** of prediction model for the occurrence risk of overall immune checkpoint inhibitor-related cardiovascular toxicity (continued)

| **No** | **Author (year)** | **Model number** | Prediction outcome | **Predictors  selection** | **Modeling methods** | **Candidate predictors** | | **Sample size (total/events number)** | | **Development cohort EPV** |
| --- | --- | --- | --- | --- | --- | --- | --- | --- | --- | --- |
|  |  |  |  |  |  | **Collection time** | **Continuous  variable  processing  method** | **Development cohort** | **External validation cohort** |  |
| 5 | Xitong Cheng  2024 | Model 1-44 | Model 1-22: The occurrence of immune-related cardiac adverse events  Model 23-44: The occurrence of severe immune-related cardiac adverse events | Model 1-11/23-33: Stepwise regression  Model 12-22/34-44: Feature importance ranking | Model 1、12、23、34：Machine learning （Logistic Regression）；Model 2、13、24、35：Machine learning （Decision Tree）；Model 3、14、25、36：Machine learning （K-Nearest Neighbor）；Model 4、15、26、37：Machine learning （Random Forest）；Model 5、16、27、38：Machine learning （Gradient Boosting Decision Tree）；Model 6、17、 28、39：Machine learning （Extreme Gradient Boosting）；Model 7、18、 29、40：Machine learning （Bootstrap Aggregating）；Model 8、19、30、41：Machine learning （Adaptive Boosting）；Model 9、20、31、42：Machine learning （Extremely Randomized Trees）；Model 10、21、32、43：Machine learning （Soft Voting Classifier）；Model 11、22、33、44：Machine learning （Hard Voting Classifier） | One month before patients received treatment | Retained as continuous variables | Model 1-22: 615/147  Model 23-44: 147/44 | None | Model 1-22: 2.88 Model 23-44: 0.86 |

Table 3-1 **Characteristics** of prediction model for the occurrence risk of overall immune checkpoint inhibitor-related cardiovascular toxicity (continued)

| **No** | **Author (year)** | **Model number** | Prediction outcome | **Predictors  selection** | **Modeling methods** | **Candidate predictors** | | **Sample size (total/events number)** | | **Development cohort EPV** |
| --- | --- | --- | --- | --- | --- | --- | --- | --- | --- | --- |
|  |  |  |  |  |  | **Collection time** | **Continuous  variable  processing  method** | **Development cohort** | **External validation cohort** |  |
| 6 | Samuel Peter Heilbroner 2021 | Model 1 | Immune-related Cardiac adverse events | Not clearly reported | Machine learning (eXtreme Gradient Boosting (XGBoost) algorithm) | 6 months before ICI treatment; 15 or 30 days post-ICI treatment | Not clearly reported | 4960/418 (training set：3937/not clearly reported；test set：1023/not clearly reported) | None | Not clearly reported |
| EPV: events per variable; ICI:immune checkpoint inhibitor. | | | | | | | | | | |

Table 3-1 **Characteristics** of prediction model for the occurrence risk of overall immune checkpoint inhibitor-related cardiovascular toxicity (continued)

| **No** | **Author (year)** | **Model number** | **Missing data** | **Prediction time** | **Final predictors** | **Model presentation** |
| --- | --- | --- | --- | --- | --- | --- |
| 1 | Zhihui Yan 2026 | Model 1 | Not clearly reported | Not clearly reported | 7（Gender；Eastern Cooperative Oncology Group performance status；Tumor stage；Complicated hypertension；Diabetes；Tumor metastasis；Systemic inflammation response index） | Nomogram |
| 2 | Chadi Ayoub 2025 | Model 1-3 | Missing data greater than 5% were dropped and the remaining missing data were encoded as 1 | Not clearly reported | Model 1:Not clearly reported. SHAP analysis identified several important predictors: (Stroke/transient ischemic attack; Ventricular tachycardia; Hypertension; Creatinine; Chronic obstructive pulmonary disease; Diabetes; Atrial fibrillation; Age > 80; Hyperlipidemia; Fam-history of coronary artery disease; Previous radiation; Smoking; Troponin; Obstructive sleep apnea; Age 60-80; Male; N-terminal pro B-type natriuretic peptide; C-reactive protein; Myocardial infarction; Lung/chest cancer)  Model 2:Not clearly reported  Model 3:Not clearly reported | Model 1:The model was explained using SHAP  Model 2:None  Model 3:None |
| 3 | Jialian Li 2025 | Model 1-3 | Not clearly reported | 30 days after initiating ICI treatment | Model 1: 6（Cardiac troponin T；Interleukin-6；T wave；Corrected QT interval using Fridericia's formula；N-terminal pro B-type natriuretic peptide；Basophil percentage）  Model 2: 10（Cardiac troponin T；Sokolow-Lyon index；Systolic blood pressure；N-terminal pro B-type natriuretic peptide；Potassium；R wave amplitude in V5 lead；QT interval；Creatine kinase-MB；PR interval；Age）  Model 3: 10（Cardiac troponin T；QT interval；Systolic blood pressure；R wave amplitude in V5 lead；S wave amplitude in V1 lead; Corrected QT interval using Fridericia's formula；Eosinophil count；QRS duration；Myoglobin；High-sensitivity C-reactive protein） | Model 1:None  Model 2:None  Model 3: The model was explained using SHAP |
| 4 | Qiuyue Li 2024 | Model 1 | Supplement missing data during collection stage | 3 treatment cycles following the initiation of immunotherapy (21 days per cycle) | 8（Hypertension；Coronary heart disease；N-terminal Probrain Natriuretic Peptide；Body mass index；Low density lipoprotein；Creatine kinase isoenzyme；α-hydroxybutyrate dehydrogenase；Combined targeted therapy） | Nomogram |

Table 3-1 **Characteristics** of prediction model for the occurrence risk of overall immune checkpoint inhibitor-related cardiovascular toxicity (continued)

| **No** | **Author (year)** | **Model number** | **Missing data** | **Prediction time** | **Final predictors** | | **Model presentation** | |
| --- | --- | --- | --- | --- | --- | --- | --- | --- |
| 5 | Xitong Cheng 2024 | Model 1-44 | Variables with missing data >50 %,a single category >90 %,or the coefficient of variation < 0.1 were deleted. The remaining missing data was filled using multivariate imputation by chained equations. | Not clearly reported | Model1-11: 11（Diabetes；Autoimmune disease；Immunomodulators；Calcium channel blockers；History of radiotherapy；Cancer type；Course of treatment；Creatine kinase MB；N-terminal B-type natriuretic peptide；C-reactive protein；Interleukin-2）  Model 12-22: 4（Interleukin-2；N-terminal B-type natriuretic peptide；C-reactive protein；Creatine kinase MB）  Model23-33:7 (Oral glucocorticoids；Morphine；Age；Left ventricular ejection fraction；N-terminal B-type natriuretic peptide；Interleukin-2；Interferon-γ)  Model 34-44: 5（N-terminal B-type natriuretic peptide；Age；Left ventricular ejection fraction；Interleukin-2；Interferon-γ） | None | |  |
| 6 | Samuel Peter Heilbroner 2021 | Model 1 | XGBoost naturally processes missing data; sensitivity analyses were conducted with multiple imputation and missing-feature removal. | Not clearly reported | 356 variables were enrolled. The SHAP summary plot displays the 40 features with the highest importance values, and a simplified model with the top 20 features was also developed.  The 40 features with the highest importance values: Not clearly reported. | The model was explained using SHAP. | |  |
| ICI:immune checkpoint inhibitor; SHAP: shapley additive explanation; XGBoost: eXtreme Gradient Boosting algorithm. | | | | | | | |  |

Table 3-2 **Characteristics** of prediction model for the occurrence risk of immune checkpoint inhibitor-related myocarditis

| **No** | **Author (year)** | **Model number** | Prediction outcome | **Predictors  selection** | **Modeling methods** | **Candidate predictors** | | **Sample size (total/events number)** | | **Development cohort EPV** |
| --- | --- | --- | --- | --- | --- | --- | --- | --- | --- | --- |
|  |  |  |  |  |  | **Collection time** | **Continuous  variable  processing  method** | **Development cohort** | **External validation cohort** |  |
| 1 | Shaqi Rao 2026 | Model 1 | ICI-associated myocarditis | Univariate/multivariate logistic regression（stepwise forward approach） | Logistic regression analysis | At admission (prior to ICI therapy) and before the onset of ICI-related myocarditis (after ICI therapy) | Retained as continuous variables | 156/58 (training set：109/43；test set:47/15) | None | 0.88 |
| 2 | Tao Luan 2026 | Model 1 | Severe ICI-associated  myocarditis | Univariate analysis、LASSO regression analysis | Logistic regression analysis | Baseline assessments at ICI initiation and prior to ICI-associated  myocarditis | Partial variables were transformed into categorical variables | 437/34 (training set：306/28；test set:131/6) | 397/31 | 1.75 |

Table 3-2 **Characteristics** of prediction model for the occurrence risk of immune checkpoint inhibitor-related myocarditis (continued)

| **No** | **Author (year)** | **Model number** | Prediction outcome | **Predictors  selection** | **Modeling methods** | **Candidate predictors** | | **Sample size (total/events number)** | | **Development cohort EPV** |
| --- | --- | --- | --- | --- | --- | --- | --- | --- | --- | --- |
|  |  |  |  |  |  | **Collection time** | **Continuous  variable  processing  method** | **Development cohort** | **External validation cohort** |  |
| 3 | Zhuoling Zheng 2026 | Model 1 | ICI-associated myocarditis | Univariate analysis and multivariate logistic  regression analysis (forward stepwise selection) | Logistic regression analysis | For myocarditis patients: before the initiation of any ICI therapy and  at the time of myocarditis diagnosis. For non-myocarditis patients: prior to the ﬁrst ICI treatment. | Not clearly reported | 196/98 | None | Not clearly reported |
| 4 | Reina Yamamoto 2026 | Model 1-2 | ICI-induced myocarditis | Pairwise pearson’s correlation matrix、chi-square statistic | Model 1: Random Forest algorithms  Model 2: Light Gradient Boosting Machine (LightGBM) algorithms | Not clearly reported | Retained as continuous variables | 48446/117 (training set：38757/94；test set:9689/23) | None | 0.16 |

Table 3-2 **Characteristics** of prediction model for the occurrence risk of immune checkpoint inhibitor-related myocarditis (continued)

| **No** | **Author (year)** | **Model number** | Prediction outcome | **Predictors  selection** | **Modeling methods** | **Candidate predictors** | | **Sample size (total/events number)** | | **Development cohort EPV** |
| --- | --- | --- | --- | --- | --- | --- | --- | --- | --- | --- |
|  |  |  |  |  |  | **Collection time** | **Continuous  variable  processing  method** | **Development cohort** | **External validation cohort** |  |
| 5 | Shanshan Li (1) 2025 | Model 1 | ICI-related myocarditis | Univariate/multivariate logistic regression（stepwise forward approach） | Logistic regression analysis | Prior to the initiation of immunotherapy | Partial variables were transformed into categorical variables | 1838/89（training set：1286/53；test set：552/36） | None | 3.12 |
| 6 | Shanshan Li (2) 2025 | Model 1-5 | ICI-related myocarditis | Univariate logistic analysis | Moedl 1：Machine learning （logistic regression）  Moedl 2：Machine learning （random forest）  Moedl 3：Machine learning （support vector machine）  Moedl 4：Machine learning （decision tree）  Moedl 5：Machine learning （gradient boosting machine） | The baseline data before receiving immunotherapy | Retained as continuous variables | 1838/89（training set：1286/53；test set：552/36） | None | 3.31 |
| EPV: events per variable; ICI:immune checkpoint inhibitor; LASSO: least absolute shrinkage and selection operator. | | | | | | | | | | |

Table 3-2 **Characteristics** of prediction model for the occurrence risk of immune checkpoint inhibitor-related myocarditis (continued)

| **No** | **Author (year)** | **Model number** | **Missing data** | **Prediction time** | **Final predictors** | **Model presentation** |
| --- | --- | --- | --- | --- | --- | --- |
| 1 | Shaqi Rao 2026 | Model 1 | Enroll patients with complete information | Not clearly reported | 5 (Body mass index; Prior tumor therapy; Prior existing disease; QRS Axis after ICIs thepy; Percent change in Standardized Sokolow-Lyon index) | Nomogram |
| 2 | Tao Luan 2026 | Model 1 | Enroll patients with complete information | Not clearly reported | 4 (Troponin; B-type natriuretic peptide; Electrocardiogram; Echocardiography) | Nomogram |
| 3 | Zhuoling Zheng 2026 | Model 1 | Enroll patients with complete information | Not clearly reported | 3 (Elevated eosinophil ratio; Reduced lymphocyte ratio; Elevated myoglobin) | None |
| 4 | Reina Yamamoto 2026 | Model 1-2 | Enroll patients with complete information | Within 3 months after initiation of ICI treatment | Model 1: A total of 89 variables were included. SHAP analysis revealed the 10 most important variables in the random forest model：（ICI combined use；Mucolytics；Antipsychotics；Lung cancer；Urologic cancer；Age；Esophageal and gastric cancer；Ipilimumab；Prescription count；Osmotically acting laxatives）  Model 2: A total of 89 variables were included. Not clearly reported | Model 1: The model was explained using SHAP  Model 2: None |
| 5 | Shanshan Li (1) 2025 | Model 1 | Not clearly reported | Not clearly reported | 5（α-hydroxybutyrate dehydrogenase；Creatine kinase-M/B；High-sensitivity cardiac troponin T；The multiple of N-terminal pro-brain natriuretic peptide；Caprini risk score） | Nomogram |
| 6 | Shanshan Li (1) 2025 | Model 1-5 | Enroll patients with complete information | Not clearly reported | 7（Electrocardiogram-score；Creatine kinase MB；High-sensitivity cardiac troponin T；N-terminal pro-B-type natriuretic peptide；Left thoracic/mediastinal radiotherapy；Hypertension；α-hydroxybutyrate dehydrogenase） | Model 2 is presented via nomogram  Model1, 3-5: None |
| ICI:immune checkpoint inhibitor; SHAP: shapley additive explanation. | | | | | | |

Table 3-3 **Characteristics** of prediction model for the occurrence risk of immune checkpoint inhibitor-related venous thromboembolism

| **No** | **Author (year)** | **Model number** | Prediction outcome | **Predictors  selection** | **Modeling methods** | **Candidate predictors** | | **Sample size (total/events number)** | | **Development cohort EPV** |
| --- | --- | --- | --- | --- | --- | --- | --- | --- | --- | --- |
|  |  |  |  |  |  | **Collection time** | **Continuous  variable  processing  method** | **Development cohort** | **External validation cohort** |  |
| 1 | Yunhan Su 2026 | Model 1 | Venous thromboembolism | Univariate analysis、 multivariate logistic regression analysis | Logistic regression analysis | Not clearly reported | Partial variables were transformed into categorical variables | 1115/105（Training set：784/68；Test set：331/37） | None | 1.94 |
| 2 | Xiaotong Xia 2025 | Model 1 | Venous thromboembolism | Univariate analysis、 multivariate logistic regression analysis | Logistic regression analysis | Not clearly reported | Not clearly reported | 1171/183（training set：967/163；test set：204/20） | None | Not clearly reported |
| 3 | Yihuai Hu 2024 | Model 1 | ICI therapy-associated venous thromboembolism | Univariate analysis、 multivariate logistic regression analysis | Logistic regression analysis | The baseline data before receiving immunotherapy | Partial variables were transformed into categorical variables | 556/31 | 392/not clearly reported | 0.56 |
| 4 | Guanzhong Liang 2024 | Model 1 | Venous thromboembolism | Univariate/multivariate logistic regression（stepwise regression approach） | Logistic regression analysis | Not clearly reported | Partial variables were transformed into categorical variables | 1373/123（training set：962/83；test set：411/40） | None | 3.46 |

Table 3-3 **Characteristics** of prediction model for the occurrence risk of immune checkpoint inhibitor-related venous thromboembolism (continued)

| **No** | **Author (year)** | **Model number** | Prediction outcome | **Predictors  selection** | **Modeling methods** | **Candidate predictors** | | **Sample size (total/events number)** | | **Development cohort EPV** |
| --- | --- | --- | --- | --- | --- | --- | --- | --- | --- | --- |
|  |  |  |  |  |  | **Collection time** | **Continuous  variable  processing  method** | **Development cohort** | **External validation cohort** |  |
| 5 | Jiarui Zhang 2024 | Model 1-3 | Venous thromboembolism | —— | —— | Not clearly reported | —— | —— | 1115/105（ICI monotherapy subgroup：152/11） | —— |
| EPV: events per variable; ICI:immune checkpoint inhibitor; ——:not applicable (not for new model development). | | | | | | | | | | |

Table 3-3 **Characteristics** of prediction model for the occurrence risk of immune checkpoint inhibitor-related venous thromboembolism (continued)

| **No** | **Author (year)** | **Model number** | **Missing data** | **Prediction time** | **Final predictors** | **Model presentation** |
| --- | --- | --- | --- | --- | --- | --- |
| 1 | Yunhan Su 2026 | Model 1 | Not clearly reported | Within 12 months after immunotherapy | 7（Eastern Cooperative Oncology Group performance status; Severe pulmonary disease; Respiratory failure; Abnormal pulmonary ventilation function; History of venous thromboembolism; Chemotherapy; Glucocorticoid therapy） | Risk score |
| 2 | Xiaotong Xia 2025 | Model 1 | Not clearly reported | 1 year after the initiation of ICIs treatment | 6（Liver cancer；Smoking；Diabetes mellitus；Liver dysfunction；History of any cardiovascular event；Khorana risk score ≥3） | Scoring system |
| 3 | Yihuai Hu 2024 | Model 1 | Random forest imputation | Not clearly reported | 5（Smoke；Heart disease；Body mass index；D-dimer；Myoglobin） | Nomogram |
| 4 | Guanzhong Liang 2024 | Model 1 | Enroll patients with complete information | Not clearly reported | 10（Age；Karnofsky performance status；Chemotherapy；Targeted therapy；White blood cell；Platelet count；Lactate dehydrogenase；Monoamine oxidase；D-dimer；Fibrinogen） | Nomogram |
| 5 | Jiarui Zhang 2024 | Model 1-3 | Not clearly reported | 12 months after the first day of ICIs treatment. | Model 1: Caprini score  Model 2: Padua score  Model 3: Khorana score | Risk Score |
| ICI:immune checkpoint inhibitor. | | | | | | |

Table 3-4 **Characteristics** of prediction model for the risk of mortality following immune checkpoint inhibitor-related cardiovascular toxicity

| **No** | **Author (year)** | **Model number** | Prediction outcome | **Predictors  selection** | **Modeling methods** | **Candidate predictors** | | **Sample size (total/events number)** | | **Development cohort EPV** |
| --- | --- | --- | --- | --- | --- | --- | --- | --- | --- | --- |
|  |  |  |  |  |  | **Collection time** | **Continuous  variable  processing  method** | **Development cohort** | **External validation cohort** |  |
| 1 | Zhihui Yan 2025 | Model 1 | Mortality after ICI treatment-related cardiovascular toxicity | Univariate/multivariate Cox regression analysis、according to literature | Cox regression analysis | Not clearly reported | Retained as continuous variables | 33/10 | None | 0.4 |
| 2 | Xiaoyun Cheng 2025 | Model 1 | All-Cause mortality after ICI-related myocarditis | Univariate Cox regression、LASSO regression analysis | Cox regression analysis | Not clearly reported | Partial variables were transformed into categorical variables | 70/25 | 40/15 | 0.89 |
| 3 | Zhengkun Guan 2025 | Model 1-2 | Model 1: The 40day overall survival of ICI myocarditis.  Model 2: The 1-year overall survival of ICI myocarditis. | Univariate Cox regression analysis，LASSO-Cox regression analysis | Cox regression analysis | Not clearly reported | Retained as continuous variables | Model 1: 90/24  Model 2: 90/40 | None | Model 1: 1.2  Model 2: 2 |
| EPV: events per variable; ICI:immune checkpoint inhibitor; LASSO: least absolute shrinkage and selection operator. | | | | | | | | | | |

Table 3-4 **Characteristics** of prediction model for the risk of mortality following immune checkpoint inhibitor-related cardiovascular toxicity (continued)

| **No** | **Author (year)** | **Model number** | **Missing data** | **Prediction time** | **Final predictors** | **Model presentation** |
| --- | --- | --- | --- | --- | --- | --- |
| 1 | Zhihui Yan 2025 | Model 1 | Not clearly reported | 120 days after the onset of cardiovascular toxicity | 4（Diabetes；Types of ICIs；Echocardiographic ventricular wall motion abnormalities；Monocyte） | Nomogram |
| 2 | Xiaoyun Cheng 2025 | Model 1 | Enroll patients with complete information | 0.5 years, 1 year, and 1.5 years after the onset of ICI-related myocarditis | 3（Combined radiotherapy；Duration of ICI treatment；Low-dose glucocorticoids in patients with severe myocarditis） | Nomogram |
| 3 | Zhengkun Guan 2025 | Model 1-2 | Enroll patients with complete information | Model 1: 40 days after the onset of ICI-associated myocarditis  Model 2: 1 year after the onset of ICI-associated myocarditis | Model 1: 3（N-terminal pro-brain natriuretic peptide；Cardiac troponin-I；Lactic dehydrogenase-to-albumin ratio）  Model 2: 4（System inflammation response index；N-terminal pro-brain natriuretic peptide；Cardiac troponin-I；Lactic dehydrogenase-to-albumin ratio） | Nomogram、web-based app |
| ICI:immune checkpoint inhibitor; app:application. | | | | | | |

Table 3-5 **Characteristics** of prediction model for the risk of major adverse cardiovascular/cardiac events following immune checkpoint inhibitor-related cardiovascular toxicity

| **No** | **Author (year)** | **Model number** | Prediction outcome | **Predictors  selection** | **Modeling methods** | **Candidate predictors** | | **Sample size (total/events number)** | | **Development cohort EPV** |
| --- | --- | --- | --- | --- | --- | --- | --- | --- | --- | --- |
|  |  |  |  |  |  | **Collection time** | **Continuous  variable  processing  method** | **Development cohort** | **External validation cohort** |  |
| 1 | John R. Power 2025 | Model 1 | Major cardiomyotoxic events after ICI myocarditis | According to literature、clinical experience、a stepwise selection procedure using Schwartz criteria | Cox regression analysis | Not clearly reported | Partial variables were transformed into categorical variables | 748/249 | Sorbonne University, Paris  (France)：35/11 Mass General Brigham,  Boston (USA)：119/49 | 9.22 |
| 2 | Zhenli Li 2024 | Model 2 | The 40‑day major adverse cardiovascular events after ICI‑related myocarditis | Univariate/multivariate COX regression、Best subset regression analysis、LASSO regression analysis | Cox regression analysis | Prior to the initiation of immunotherapy and at the onset of myocarditis | Retained as continuous variables | 77/not clearly reported | None | Not clearly reported |

Table 3-5 **Characteristics** of prediction model for the risk of major adverse cardiovascular/cardiac events following immune checkpoint inhibitor-related cardiovascular toxicity (continued)

| **No** | **Author (year)** | **Model number** | Prediction outcome | **Predictors  selection** | **Modeling methods** | **Candidate predictors** | | **Sample size (total/events number)** | | **Development cohort EPV** |
| --- | --- | --- | --- | --- | --- | --- | --- | --- | --- | --- |
|  |  |  |  |  |  | **Collection time** | **Continuous  variable  processing  method** | **Development cohort** | **External validation cohort** |  |
| 3 | Rui Lu 2024 | Model 1-5 | Major adverse cardiovascular events following ICI-associated myocarditis | Model 1: Univariate analysis、multivariate logistic regression analysis  Model 2-5: Univariate logistic analysis | Model 1: Logistic regression analysis  Model 2: Machine learning (Logistic Regression algorithm）  Model 3: Machine learning (Random Forest algorithm）  Model 4: Machine learning (eXtreme Gradient Boosting (XGBoost) algorithm)  Model 5: Machine learning (Light Gradient Boosting Machine) | Not clearly reported | Partial variables were transformed into categorical variables | 35/17 | None | Not clearly reported |
| EPV: events per variable; ICI:immune checkpoint inhibitor; LASSO: least absolute shrinkage and selection operator. | | | | | | | | | | |

Table 3-5 **Characteristics** of prediction model for the risk of major adverse cardiovascular/cardiac events following immune checkpoint inhibitor-related cardiovascular toxicity (continued)

| **No** | **Author (year)** | **Model number** | **Missing data** | **Prediction time** | **Final predictors** | **Model presentation** |
| --- | --- | --- | --- | --- | --- | --- |
| 1 | John R. Power 2025 | Model 1 | Multiple imputation | 30 days after clinical presentation with ICI myocarditis | 5（Active thymoma；Presence of cardiomuscular symptoms；Low Sokolow-Lyon QRS voltage on presenting electrocardiogram；Left ventricular ejection fraction；Elevated troponin） | Risk Score |
| 2 | Zhenli Li 2024 | Model 2 | Not clearly reported | 40 days after the onset of ICI-associated myocarditis | 2（Neutrophil-to-high-density lipoprotein cholesterol ratio and Lactate dehydrogenase-to-albumin ratio at the onset of myocarditis） | Nomogram、web-based app |
| 3 | Rui Lu 2024 | Model 1-5 | Enroll patients with complete information | Not clearly reported | Model 1: 2（Neutrophil to lymphocyte ratio；ST‑T segment changes）  Model 2-5: 6 (ST‑T segment changes; Second-to third-degree atrioventricular block; Ventricular tachycardia; Left ventricular ejection fraction≤50%; White blood cell count; Neutrophil to lymphocyte ratio) | Model 1:Regression Formula、nomogram  Model 2、4-5：None  Model 3: The model was explained using SHAP |
| ICI:immune checkpoint inhibitor; app:application; SHAP: shapley additive explanation. | | | | | | |

Table 3-6 **Characteristics** of diagnostic prediction model for grading the severity of immune checkpoint inhibitor-related cardiovascular toxicity

| **No** | **Author (year)** | **Model number** | Prediction outcome | **Predictors  selection** | **Modeling methods** | **Candidate predictors** | | **Sample size (total/events number)** | | **Development cohort EPV** |
| --- | --- | --- | --- | --- | --- | --- | --- | --- | --- | --- |
|  |  |  |  |  |  | **Collection time** | **Continuous  variable  processing  method** | **Development cohort** | **External validation cohort** |  |
| 1 | Zhuoling Zheng  2026 | Model 2 | The severity of ICI-associated myocarditis | Univariate analysis and multivariate logistic  regression analysis (forward stepwise selection) | Logistic regression analysis | For myocarditis patients: before the initiation of any ICI therapy and  at the time of myocarditis diagnosis. | Not clearly reported | 98/27 | None | Not clearly reported |
| 2 | Zhenli Li 2024 | Model 1 | The severity of ICI‑related myocarditis | Univariate/bivariate logistic analysis、receiver operating curves analysis | Logistic regression analysis | Prior to the initiation of immunotherapy and at the onset of myocarditis | Retained as continuous variables | 77/31 | None | 2.58 |
| EPV: events per variable; ICI:immune checkpoint inhibitor. | | | | | | | | | | |

Table 3-6 **Characteristics** of diagnostic prediction model for grading the severity of immune checkpoint inhibitor-related cardiovascular toxicity (continued)

| **No** | **Author (year)** | **Model number** | **Missing data** | **Prediction time** | **Final predictors** | **Model presentation** |
| --- | --- | --- | --- | --- | --- | --- |
| 1 | Zhuoling Zheng  2026 | Model 2 | Enroll patients with complete information | At the onset of ICI-associated myocarditis | 3 (T-wave changes; Complete/incomplete bundle branch block; Creatine kinase elevation) | None |
| 2 | Zhenli Li 2024 | Model 1 | Not clearly reported | At the onset of ICI-associated myocarditis | 2（Lactate dehydrogenase-to-albumin ratio（LAR） at the onset of myocarditis；the change rate of LAR from baseline to the occurrence of myocarditis） | Nomogram、web-based app |
| ICI:immune checkpoint inhibitor; app:application. | | | | | | |

Table 4-1 Performance of prediction model for the occurrence risk of overall immune checkpoint inhibitor-related cardiovascular toxicity

| **No** | **Author (year)** | **Model number** | **Apparent performance** | **Internal validation performance** | **External validation performance** |
| --- | --- | --- | --- | --- | --- |
| 1 | Zhihui Yan 2026 | Model 1 | None | Based on bootstrap: AUC-ROC：0.77（95% CI：0.70–0.85）; accuracy：0.73 (95% CI：0.66–0.80); sensitivity：0.78 (95% CI：0.70–0.85); specificity：0.65 (95% CI：0.53–0.77); positive predictive value：0.82 (95% CI：0.74–0.89); negative predictive value：0.59 (95% CI：0.47–0.71). Hosmer-Lemeshow：*P*＝0.053，the calibration curve indicated that there is a good agreement between the predictive probability of the model and the actual situation. DCA revealed that this model has a better effect on the clinical benefit. | None |
| 2 | Chadi Ayoub  2025 | Model 1 | None | Based on splitting:  Accuracy: 0.72 (95% CI：0.691-0.72); AUC-ROC: 0.645 (95% CI：0.63- 0.64); Negative predictive value: 0.9769 (95% CI：0.96-0.98); True positive rate: 0.5714 (95% CI：0.562-0.58). | None |
|  |  | Model 2 | None | Based on splitting:  Accuracy: 0.5519 (95% CI：0.542-0.556); AUC-ROC: 0.595 (95% CI：0.57-0.6); Negative predictive value: 0.9653 (95% CI：0.96-0.97); True positive rate: 0.50 (95% CI：0.492-0.504). | None |
|  |  | Model 3 | None | Based on splitting:  Accuracy: 0.6913 (95% CI：0.681-0.70); AUC-ROC: 0.717 (95% CI：0.70- 0.72); Negative predictive value: 0.98 (95% CI：0.97-0.98); True positive rate: 0.6428 (95% CI：0.6343-0.643). | None |

Table 4-1 Performance of prediction model for the occurrence risk of overall immune checkpoint inhibitor-related cardiovascular toxicity (continued)

| **No** | **Author (year)** | **Model number** | **Apparent performance** | **Internal validation performance** | **External validation performance** |
| --- | --- | --- | --- | --- | --- |
| 3 | Jialian Li 2025 | Model 1 | None | Based on splitting+bootstrap: Training set: AUC-ROC after bootstrapping: 0.60. Test set: AUC-ROC: 0.80 (95% CI: 0.799–0.806); sensitivity: 0.05; F1 score: 0.09; accuracy: 0.83; positive predictive value: 1; negative predictive value: 0.83. | None |
|  |  | Model 2 | None | Based on splitting+bootstrap: Training set: AUC-ROC after bootstrapping: 0.93. Test set: AUC-ROC: 0.74 (95% CI: 0.736–0.745); sensitivity: 0.55; F1 score: 0.67; accuracy: 0.90; positive predictive value: 0.86; negative predictive value: 0.91. | None |
|  |  | Model 3 | None | Based on splitting+bootstrap: Training set: AUC-ROC after bootstrapping: 0.92. Test set: AUC-ROC: 0.83 (95% CI: 0.827–0.834): sensitivity: 0.59; F1 score: 0.68; accuracy: 0.90: positive predictive value: 0.81: negative predictive value: 0.92. DCA demonstrated that compared to the model 1 and model 2, the model 3 provided a greater net benefit; CIC demonstrated its net benefit across a broad range of threshold probabilities. Calibration slope：0.906. | None |

Table 4-1 Performance of prediction model for the occurrence risk of overall immune checkpoint inhibitor-related cardiovascular toxicity (continued)

| **No** | **Author (year)** | **Model number** | **Apparent performance** | **Internal validation performance** | **External validation performance** |
| --- | --- | --- | --- | --- | --- |
| 4 | Qiuyue Li 2024 | Model 1 | AUC-ROC：0.935 (95%CI：0.899-0.971). The calibration curve and Hosmer-Lemeshow test indicated good model fitting performance (Hosmer-Lemeshow test: *P* = 0.60). DCA revealed that the model had a broad optimal threshold range and certain clinical utility. | None | None |
| 5 | Xitong Cheng  2024 | Model 1 | None | Based on cross validation：  Accuracy：0.8553；Precision：0.7960；Recall：0.5639；F1 score：0.6452；AUC-ROC：0.7552；AUPR：0.7320.  Brier Score：0.1447. | None |
|  |  | Model 2 | None | Based on cross validation：  Accuracy：0.8537；Precision：0.8194；Recall：0.4674；F1 score：0.5888；AUC-ROC：0.7209；AUPR：0.7068.  Brier Score：0.1463. | None |
|  |  | Model 3 | None | Based on cross validation：  Accuracy：0.8569；Precision：0.8266；Recall：0.5239；F1 score：0.6355；AUC-ROC：0.7428；AUPR：0.7322.  Brier Score：0.1431. | None |
|  |  | Model 4 | None | Based on cross validation：  Accuracy：0.9024；Precision：0.8825；Recall：0.6926；F1 score：0.7698；AUC-ROC：0.8303；AUPR：0.8242.  Brier Score：0.0976. | None |

Table 4-1 Performance of prediction model for the occurrence risk of overall immune checkpoint inhibitor-related cardiovascular toxicity (continued)

| **No** | **Author (year)** | **Model number** | **Apparent performance** | **Internal validation performance** | **External validation performance** |
| --- | --- | --- | --- | --- | --- |
| 5 | Xitong Cheng  2024 | Model 5 | None | Based on cross validation：  Accuracy：0.8130；Precision：0.7299；Recall：0.7062；F1 score：0.6832；AUC-ROC：0.7758；AUPR：0.7530.  Brier Score：0.1870. | None |
|  |  | Model 6 | None | Based on cross validation：  Accuracy：0.8927；Precision：0.8514；Recall：0.7133；F1 score：0.7627；AUC-ROC：0.8309；AUPR：0.8165.  Brier Score：0.1073. | None |
|  |  | Model 7 | None | Based on cross validation：  Accuracy：0.8293；Precision：0.8357；Recall：0.6646；F1 score：0.6853；AUC-ROC：0.7721；AUPR：0.7900.  Brier Score：0.1707. | None |
|  |  | Model 8 | None | Based on cross validation：  Accuracy：0.8780；Precision：0.8128；Recall：0.6529；F1 score：0.7211；AUC-ROC：0.8008；AUPR：0.7743.  Brier Score：0.1220. | None |
|  |  | Model 9 | None | Based on cross validation：  Accuracy：0.7919；Precision：0.7631；Recall：0.7191；F1 score：0.6839；AUC-ROC：0.7661；AUPR：0.7744.  Brier Score：0.2081. | None |

Table 4-1 Performance of prediction model for the occurrence risk of overall immune checkpoint inhibitor-related cardiovascular toxicity (continued)

| **No** | **Author (year)** | **Model number** | **Apparent performance** | **Internal validation performance** | **External validation performance** |
| --- | --- | --- | --- | --- | --- |
| 5 | Xitong Cheng  2024 | Model 10 | None | Based on cross validation：  Accuracy：0.9089；Precision：0.9309；Recall：0.6862；F1 score：0.7817；AUC-ROC：0.8324；AUPR：0.8460.  Brier Score：0.0911. | None |
|  |  | Model 11 | None | Based on cross validation：  Accuracy：0.8943；Precision：0.8766；Recall：0.6791；F1 score：0.7536；AUC-ROC：0.8202；AUPR：0.8161.  Brier Score：0.1057. | None |
|  |  | Model 12 | None | Based on cross validation：  Accuracy：0.8780；Precision：0.9110；Recall：0.5444；F1 score：0.6774；AUC-ROC：0.7636；AUPR：0.7821.  Brier Score：0.1220. | None |
|  |  | Model 13 | None | Based on cross validation：  Accuracy：0.8569；Precision：0.8545；Recall：0.5101；F1 score：0.6303；AUC-ROC：0.7379；AUPR：0.7409.  Brier Score：0.1431. | None |
|  |  | Model 14 | None | Based on cross validation：  Accuracy：0.8585；Precision：0.8256；Recall：0.5166；F1 score：0.6346；AUC-ROC：0.7412；AUPR：0.7288.  Brier Score：0.1415. | None |

Table 4-1 Performance of prediction model for the occurrence risk of overall immune checkpoint inhibitor-related cardiovascular toxicity (continued)

| **No** | **Author (year)** | **Model number** | **Apparent performance** | **Internal validation performance** | **External validation performance** |
| --- | --- | --- | --- | --- | --- |
| 5 | Xitong Cheng  2024 | Model 15 | None | Based on cross validation：  Accuracy：0.9008；Precision：0.8766；Recall：0.6805；F1 score：0.7640；AUC-ROC：0.8253；AUPR：0.8167.  Brier Score：0.0992. | None |
|  |  | Model 16 | None | Based on cross validation：  Accuracy：0.8976；Precision：0.8811；Recall：0.6605；F1 score：0.7531；AUC-ROC：0.8163；AUPR：0.8114.  Brier Score：0.1024. | None |
|  |  | Model 17 | None | Based on cross validation：  Accuracy：0.8813；Precision：0.8089；Recall：0.6600；F1 score：0.7248；AUC-ROC：0.8055；AUPR：0.7751.  Brier Score：0.1187. | None |
|  |  | Model 18 | None | Based on cross validation：  Accuracy：0.8911；Precision：0.8857；Recall：0.6264；F1 score：0.7312；AUC-ROC：0.8004；AUPR：0.8008.  Brier Score：0.1089. | None |
|  |  | Model 19 | None | Based on cross validation：  Accuracy：0.9122；Precision：0.9448；Recall：0.6736；F1 score：0.7845；AUC-ROC：0.8303；AUPR：0.8482.  Brier Score：0.0878. | None |

Table 4-1 Performance of prediction model for the occurrence risk of overall immune checkpoint inhibitor-related cardiovascular toxicity (continued)

| **No** | **Author (year)** | **Model number** | **Apparent performance** | **Internal validation performance** | **External validation performance** |
| --- | --- | --- | --- | --- | --- |
| 5 | Xitong Cheng  2024 | Model 20 | None | Based on cross validation：  Accuracy：0.8927；Precision：0.8456；Recall：0.6736；F1 score：0.7481；AUC-ROC：0.8176；AUPR：0.7986.  Brier Score：0.1073. | None |
|  |  | Model 21 | None | Based on cross validation：  Accuracy：0.9154；Precision：0.9323；Recall：0.7007；F1 score：0.7985；AUC-ROC：0.8418；AUPR：0.8523.  Brier Score：0.0846. | None |
|  |  | Model 22 | None | Based on cross validation：  Accuracy：0.9057；Precision：0.9245；Recall：0.6595；F1 score：0.7694；AUC-ROC：0.8212；AUPR：0.8327.  Brier Score：0.0943. | None |
|  |  | Model 23 | None | Based on cross validation：  Accuracy：0.7269；Precision：0.6913；Recall：0.5889；F1 score：0.5475；AUC-ROC：0.6856；AUPR：0.7008.  Brier Score：0.2731. | None |
|  |  | Model 24 | None | Based on cross validation：  Accuracy：0.7411；Precision：0.2533；Recall：0.3278；F1 score：0.2857；AUC-ROC：0.6258；AUPR：0.6929.  Brier Score：0.2589. | None |

Table 4-1 Performance of prediction model for the occurrence risk of overall immune checkpoint inhibitor-related cardiovascular toxicity (continued)

| **No** | **Author (year)** | **Model number** | **Apparent performance** | **Internal validation performance** | **External validation performance** |
| --- | --- | --- | --- | --- | --- |
| 5 | Xitong Cheng  2024 | Model 25 | None | Based on cross validation：  Accuracy：0.6648；Precision：0.5855；Recall：0.5222；F1 score：0.4864；AUC-ROC：0.6228；AUPR：0.6250.  Brier Score：0.3352. | None |
|  |  | Model 26 | None | Based on cross validation：  Accuracy：0.7754；Precision：0.8329；Recall：0.5472；F1 score：0.5731；AUC-ROC：0.7091；AUPR：0.7575.  Brier Score：0.2246. | None |
|  |  | Model 27 | None | Based on cross validation：  Accuracy：0.7945；Precision：0.7711；Recall：0.7278；F1 score：0.7054；AUC-ROC：0.7744；AUPR：0.7901.  Brier Score：0.2055. | None |
|  |  | Model 28 | None | Based on cross validation：  Accuracy：0.8218；Precision：0.7729；Recall：0.7750；F1 score：0.7397；AUC-ROC：0.8082；AUPR：0.8078.  Brier Score：0.1782. | None |
|  |  | Model 29 | None | Based on cross validation：  Accuracy：0.7609；Precision：0.7707；Recall：0.6861；F1 score：0.6360；AUC-ROC：0.7390；AUPR：0.7755.  Brier Score：0.2391. | None |

Table 4-1 Performance of prediction model for the occurrence risk of overall immune checkpoint inhibitor-related cardiovascular toxicity (continued)

| **No** | **Author (year)** | **Model number** | **Apparent performance** | **Internal validation performance** | **External validation performance** |
| --- | --- | --- | --- | --- | --- |
| 5 | Xitong Cheng  2024 | Model 30 | None | Based on cross validation：  Accuracy：0.7538；Precision：0.7418；Recall：0.5139；F1 score：0.5304；AUC-ROC：0.6822；AUPR：0.6992.  Brier Score：0.2462. | None |
|  |  | Model 31 | None | Based on cross validation：  Accuracy：0.8425；Precision：0.8456；Recall：0.7750；F1 score：0.7667；AUC-ROC：0.8230；AUPR：0.8442.  Brier Score：0.1575. | None |
|  |  | Model 32 | None | Based on cross validation：  Accuracy：0.8359；Precision：0.8456；Recall：0.7528；F1 score：0.7496；AUC-ROC：0.8119；AUPR：0.8364.  Brier Score：0.1641. | None |
|  |  | Model 33 | None | Based on cross validation：  Accuracy：0.8292；Precision：0.8456；Recall：0.7306；F1 score：0.7298；AUC-ROC：0.8008；AUPR：0.8286.  Brier Score：0.1708. | None |
|  |  | Model 34 | None | Based on cross validation：  Accuracy：0.8710；Precision：0.8445；Recall：0.7028；F1 score：0.7610；AUC-ROC：0.8221；AUPR：0.8177.  Brier Score：0.1290. | None |

Table 4-1 Performance of prediction model for the occurrence risk of overall immune checkpoint inhibitor-related cardiovascular toxicity (continued)

| **No** | **Author (year)** | **Model number** | **Apparent performance** | **Internal validation performance** | **External validation performance** |
| --- | --- | --- | --- | --- | --- |
| 5 | Xitong Cheng  2024 | Model 35 | None | Based on cross validation：  Accuracy：0.7260；Precision：0.5932；Recall：0.7694；F1 score：0.6456；AUC-ROC：0.7371；AUPR：0.7152.  Brier Score：0.2740. | None |
|  |  | Model 36 | None | Based on cross validation：  Accuracy：0.7885；Precision：0.7447；Recall：0.6833；F1 score：0.6603；AUC-ROC：0.7571；AUPR：0.7612.  Brier Score：0.2115. | None |
|  |  | Model 37 | None | Based on cross validation：  Accuracy：0.8566；Precision：0.7964；Recall：0.7472；F1 score：0.7572；AUC-ROC：0.8241；AUPR：0.8092.  Brier Score：0.1434. | None |
|  |  | Model 38 | None | Based on cross validation：  Accuracy：0.8290；Precision：0.8000；Recall：0.6778；F1 score：0.7011；AUC-ROC：0.7841；AUPR：0.7865.  Brier Score：0.1710. | None |
|  |  | Model 39 | None | Based on cross validation：  Accuracy：0.8152；Precision：0.7889；Recall：0.7000；F1 score：0.7003；AUC-ROC：0.7802；AUPR：0.7886.  Brier Score：0.1848. | None |

Table 4-1 Performance of prediction model for the occurrence risk of overall immune checkpoint inhibitor-related cardiovascular toxicity (continued)

| **No** | **Author (year)** | **Model number** | **Apparent performance** | **Internal validation performance** | **External validation performance** |
| --- | --- | --- | --- | --- | --- |
| 5 | Xitong Cheng  2024 | Model 40 | None | Based on cross validation：  Accuracy：0.8361；Precision：0.8143；Recall：0.6778；F1 score：0.7141；AUC-ROC：0.7891；AUPR：0.7936.  Brier Score：0.1639. | None |
|  |  | Model 41 | None | Based on cross validation：  Accuracy：0.8568；Precision：0.8048；Recall：0.7278；F1 score：0.7540；AUC-ROC：0.8194；AUPR：0.8070.  Brier Score：0.1432. | None |
|  |  | Model 42 | None | Based on cross validation：  Accuracy：0.8359；Precision：0.8341；Recall：0.7250；F1 score：0.7356；AUC-ROC：0.8025；AUPR：0.8202.  Brier Score：0.1641. | None |
|  |  | Model 43 | None | Based on cross validation：  Accuracy：0.8841；Precision：0.8521；Recall：0.7722；F1 score：0.8004；AUC-ROC：0.8513；AUPR：0.8461.  Brier Score：0.1159. | None |
|  |  | Model 44 | None | Based on cross validation：  Accuracy：0.8708；Precision：0.8521；Recall：0.7278；F1 score：0.7721；AUC-ROC：0.8291；AUPR：0.8305.  Brier Score：0.1292. | None |

Table 4-1 Performance of prediction model for the occurrence risk of overall immune checkpoint inhibitor-related cardiovascular toxicity (continued)

| **No** | **Author (year)** | **Model number** | **Apparent performance** | **Internal validation performance** | **External validation performance** |
| --- | --- | --- | --- | --- | --- |
| 6 | Samuel Peter Heilbroner  2021 | Model 1 | None | Based on splitting: When all 356 variables were included in the model, the AUC-ROC value varied between 0.63 and 0.72 as the time window for predictions changed. The AUC-ROC value at the 100-day time window was 0.65 (95% CI: 0.58-0.75) and the C statistic value was 0.66 (95% CI: 0.57-0.71).  When the model only retained the top 20 variables ranked by SHAP feature importance, both the AUC-ROC and C statistic were 0.65. | None |
| AUC-ROC: area under receiver operating curves; CI:confidence interval; DCA: decision curve analysis ; CIC: clinical impact curve. | | | | | |

Table 4-2 Performance of prediction model for the occurrence risk of immune checkpoint inhibitor-related myocarditis

| **No** | **Author (year)** | **Model number** | **Apparent performance** | **Internal validation performance** | **External validation performance** |
| --- | --- | --- | --- | --- | --- |
| 1 | Shaqi Rao 2026 | Model 1 | None | Based on splitting+bootstrap: Training set: AUC-ROC：0.93（95% CI：0.88-0.98）.  Calibration curve closely overlaps the ideal reference line.  DCA demonstrated that this nomogram model yields satisfactory clinical net benefit.  Test set: AUC-ROC：0.89（95% CI：0.79-0.99）.  Calibration curve closely overlaps the ideal reference line.  DCA demonstrated that this nomogram model yields satisfactory clinical net benefit. | None |
| 2 | Tao Luan  2026 | Model 1 | AUC-ROC：0.926 (95% CI: 0.880-0.971).  Calibration curves exhibited good agreement.  DCA showed that the nomogram yielded a positive net benefit over the‘treat-all’ and‘treat-none’ strategies across clinically relevant threshold probabilities, supporting potential clinical utility and informing threshold selection in practice (threshold probability: 0.00-0.62). | Based on splitting:  AUC-ROC：0.885 (95% CI: 0.742-1.000).  Calibration curves exhibited good agreement.  DCA showed that the nomogram yielded a positive net benefit over the‘treat-all’ and‘treat-none’ strategies across clinically relevant threshold probabilities, supporting potential clinical utility and informing threshold selection in practice (threshold probability: 0.16-0.96). | Based on Geographic analysis:  AUC-ROC：0.949 (95% CI: 0.884-1.000).  Calibration curves exhibited good agreement.  DCA showed that the nomogram yielded a positive net benefit over the‘treat-all’ and‘treat-none’ strategies across clinically relevant threshold probabilities, supporting potential clinical utility and informing threshold selection in practice (threshold probability: 0.16-0.96). |

Table 4-2 Performance of prediction model for the occurrence risk of immune checkpoint inhibitor-related myocarditis (continued)

| **No** | **Author (year)** | **Model number** | **Apparent performance** | **Internal validation performance** | **External validation performance** |
| --- | --- | --- | --- | --- | --- |
| 3 | Zhuoling Zheng  2026 | Model 1 | AUC-ROC：0.699 (95% CI: 0.626-0.772). | Based on bootstrap: AUC-ROC: 95% CI: 0.635-0.762. | None |
| 4 | Reina Yamamoto 2026 | Model 1 | None | Based on splitting+cross-validation: Training set: (Following 100 iterations of threefold cross-validation): AUC-ROC：0.699±0.009；F5 score：0.160±0.013；Accuracy：0.916±0.028；Recall：0.355±0.050；Precision：0.012±0.002；Average Precision：0.009±0.001. Test set：AUC-ROC：0.634 (95% CI: 0.507–0.751)；Accuracy：0.974 (95% CI: 0.971–0.977)；F5 score：0.191 (95% CI: 0.189–0.193)；Recall：0.260 (95% CI: 0.055–0.407)；Precision：0.025 (95% CI: 0.004–0.040). | None |
|  |  | Model 2 | None | Based on splitting+cross-validation: Training set：(Following 100 iterations of threefold cross-validation): AUC-ROC：0.609±0.024；F5 score：0.103±0.011；Accuracy：0.797±0.080；Recall：0.403±0.101；Precision：0.006±0.002；Average Precision：0.005±0.002. Test set：AUC-ROC：0.635 (95% CI: 0.493–0.754)；Accuracy：0.969 (95% CI: 0.966–0.972)；F5 score：0.178 (95% CI: 0.176–0.180)；Recall：0.260 (95% CI: 0.056–0.754)；Precision：0.020 (95% CI: 0.004–0.034). | None |

Table 4-2 Performance of prediction model for the occurrence risk of immune checkpoint inhibitor-related myocarditis (continued)

| **No** | **Author (year)** | **Model number** | **Apparent performance** | **Internal validation performance** | **External validation performance** |
| --- | --- | --- | --- | --- | --- |
| 5 | Shanshan Li (1) 2025 | Model 1 | ROC-AUC：0.831 (95% CI: 0.772–0.890); accuracy：0.837 (95% CI: 0.815–0.857); sensitivity：0.842 (95% CI: 0.821–0.862); specificity：0.717 (95% CI: 0.596–0.838)；youden Index：0.559. | Based on splitting: AUC-ROC：0.844 (95% CI: 0.786–0.903). Calibration plot indicated a close alignment between the predicted and standard curves. Hosmer-Lemeshow test: *P*=0.093. | None |
| 6 | Shanshan Li (2) 2025 | Model 1 | Accuracy：0.864. | Based on splitting: Accuracy：0.877；Recall：0.861；Precision：0.330；Jaccard coefficient：0.313；F1 score：0.477；AUC-ROC：0.923 (95% CI: 0.864-0.982). | None |
|  |  | Model 2 | Accuracy：0.941. | Based on splitting: Accuracy：0.981；Recall：0.833；Precision：0.395；Jaccard coefficient：0.366；F1 score：0.536；AUC-ROC：0.942 (95% CI: 0.896-0.987).  Hosmer-Lemeshow test: *P*=0.809. | None |
|  |  | Model 3 | Accuracy：0.940. | Based on splitting: Accuracy：0.911；Recall：0.250；Precision：0.290；Jaccard coefficient：0.155；F1 score：0.269；AUC-ROC：0.850 (95% CI: 0.767-0.932). | None |
|  |  | Model 4 | Accuracy：0.867. | Based on splitting: Accuracy：0.873；Recall：0.861；Precision：0.323；Jaccard coefficient：0.307；F1 score：0.470；AUC-ROC：0.869 (95% CI: 0.807-0.931). | None |
|  |  | Model 5 | Accuracy：0.927. | Based on splitting: Accuracy：0.922；Recall：0.806；Precision：0.446；Jaccard coefficient：0.403；F1 score：0.574；AUC-ROC：0.936 (95% CI: 0.886-0.985). | None |
| AUC-ROC: area under receiver operating curves; CI:confidence interval; DCA: decision curve analysis. | | | | | |

Table 4-3 Performance of prediction model for the occurrence risk of immune checkpoint inhibitor-related venous thromboembolism

| **No** | **Author (year)** | **Model number** | **Apparent performance** | **Internal validation performance** | **External validation performance** |
| --- | --- | --- | --- | --- | --- |
| 1 | Yunhan Su 2026 | Model 1 | AUC-ROC：0.822 (95% CI 0.762-0.882). Hosmer-Lemeshow：*P*=0.635. | Based on splitting: AUC-ROC：0.779 (95% CI 0.687-0.871). Hosmer-Lemeshow：*P*=0.553. | None |
| 2 | Xiaotong Xia 2025 | Model 1 | None | Based on splitting: AUC-ROC：0.818 (95% CI: 0.686–0.950). | None |
| 3 | Yihuai Hu 2024 | Model 1 | AUC-ROC: 0.828 (95%CI：0.740–0.916); sensitivity：71.0%; specificity：87.4%. DCA and CIC revealed the model provided valuable references for clinical decision-making. | Based on bootstrap: The calibration curve was close to the reference line, suggesting good consistency between the predicted probability and observed probability. | Based on temporal phase analysis: AUC-ROC:0.794 (95% CI: 0.647-0.941). The calibration curve was close to the reference line, suggesting good consistency between the predicted probability and observed probability. DCA revealed that the model provided certain reference value for clinical decision-making. |
| 4 | Guanzhong Liang 2024 | Model 1 | AUC-ROC：0.815 (95% CI: 0.772–0.858). Brier scores：0.068. Hosmer and Lemeshow tests：*p*=0.154; Calibration curve showed good agreement between predicted and observed outcomes. DCA revealed that the predictive model offers a substantial net benefit across the probability thresholds of 0.01–0.47 for the training set;  CIC revealed that the predictive model offers a substantial net benefit across certain probability thresholds. | Based on splitting: AUC-ROC：0.753 (95% CI：0.672– 0.835). Brier scores：0.080. Hosmer Lemeshow tests：*p*=0.292;  Calibration curve showed good agreement between predicted and observed outcomes. DCA revealed that the predictive model offers a substantial net benefit across the probability thresholds of 0.01–0.56 for the validation set; CIC revealed that the predictive model offers a substantial net benefit across certain probability thresholds. | None |

Table 4-3 Performance of prediction model for the occurrence risk of immune checkpoint inhibitor-related venous thromboembolism (continued)

| **No** | **Author (year)** | **Model number** | **Apparent performance** | **Internal validation performance** | **External validation performance** |
| --- | --- | --- | --- | --- | --- |
| 5 | Jiarui Zhang 2024 | Model 1 | None | None | ICI-treated patient group：AUC-ROC: 0.743 (95% CI: 0.688 - 0.799). ICI monotherapy group：AUC-ROC: 0.762 (95% CI: 0.583–0.942). |
|  |  | Model 2 | None | None | ICI-treated patient group：AUC-ROC: 0.745 (95% CI: 0.687 ‐ 0.803). ICI monotherapy group：AUC-ROC: 0.656 (95% CI 0.458-0.854). |
|  |  | Model 3 | None | None | ICI-treated patient group：AUC-ROC: 0.553 (95% CI, 0.493 ‐ 0.613). ICI monotherapy group：AUC-ROC: 0.549 (95% CI 0.367‐0.731). |
| AUC-ROC: area under receiver operating curves; CI:confidence interval; DCA: decision curve analysis ; CIC: clinical impact curve; ICI: immune checkpoint inhibitor. | | | | | |

Table 4-4 Performance of prediction model for the risk of mortality following immune checkpoint inhibitor-related cardiovascular toxicity

| **No** | **Author (year)** | **Model number** | **Apparent performance** | **Internal validation performance** | **External validation performance** |
| --- | --- | --- | --- | --- | --- |
| 1 | Zhihui Yan 2025 | Model 1 | AUC-ROC：0.88 (95% CI ：0.75-1.00). The calibration curve showed that the predicted curve was well fitted with the observed curve, indicating satisfactory calibration performance. DCA demonstrated that the model achieved favorable clinical benefit. | None | None |
| 2 | Xiaoyun Cheng 2025 | Model 1 | None | Based on bootstrap: The time-dependent AUC-ROC: AUC-ROC: 180 days=0.832 (95% CI：0.687~0.935); AUC-ROC: 360 days=0.835 (95% CI：0.644~0.927); AUC-ROC: 540 days=0.924 (95% CI：0.700~0.991). Calibration plots demonstrated that the constructed nomogram model aligns well with the actual follow-up outcomes. DCA results demonstrated that the net benefit curves outperformed the “all-population assumption” at different time points. | Based on temporal phase analysis: The time-dependent AUC-ROC: AUC-ROC: 180 days: 0.821 (95% CI：0.669-0.972); AUC-ROC: 360 days: 0.806 (95% CI：0.659-0.954); AUC-ROC: 540 days: 0.789 (95% CI：0.594-0.985). Calibration plots demonstrated that the constructed nomogram model aligns well with the actual follow-up outcomes.  DCA results demonstrated that the net benefit curves outperformed the “all-population assumption” at different time points. |
| 3 | Zhengkun Guan 2025 | Model 1 | AUC-ROC：0.867 (95% CI: 0.774−0.960); C statistic：0.824. Brier score：0.12. DCA indicates that when the threshold probability ranges from 10% to 80%, the application of the model yields more benefits. | Based on bootstrap: C statistic：0.812. Calibration curve illustrates a great concordance between the model’s predictions and the actual observations. | None |

Table 4-4 Performance of prediction model for the risk of mortality following immune checkpoint inhibitor-related cardiovascular toxicity (continued)

| **No** | **Author (year)** | **Model number** | **Apparent performance** | **Internal validation performance** | **External validation performance** |
| --- | --- | --- | --- | --- | --- |
| 3 | Zhengkun Guan 2025 | Model 2 | AUC-ROC: 0.765 (95% CI: 0.664−0.866); C statistic:0.742. Brier score：0.18. DCA reveals that when the threshold probability ranges from 35% to 87%, the application of the model added more benefits. | Based on bootstrap: C statistic：0.728. Calibration curve shows a great concordance between the model’s predictions and the actual observations. | None |
| AUC-ROC: area under receiver operating curves; CI:confidence interval; DCA: decision curve analysis. | | | | | |

Table 4-5 Performance of prediction model for the risk of major adverse cardiovascular/cardiac events following immune checkpoint inhibitor-related cardiovascular toxicity

| **No** | **Author (year)** | **Model number** | **Apparent performance** | **Internal validation performance** | **External validation performance** |
| --- | --- | --- | --- | --- | --- |
| 1 | John R. Power 2025 | Model 1 | None | Based on 10-fold cross-validation: C statistic of 0.70 (95% CI: 0.67–0.73) on Day 1 and remained unchanged at 0.71 (95% CI: 0.66–0.75) by Day 3. | In the Mass General Brigham cohort, the risk score had a C statistic of 0.63 (95% CI: 0.56–0.71) on Day 1 which increased to 0.72 (95% CI: 0.62–0.80) on Day 2 and to 0.75 (95% CI: 0.65–0.82) on Day 3. In the Sorbonne University cohort, the risk score had a C statistic of 0.75 (95% CI: 0.53–0.86) on Day 1 and 0.74 (95% CI: 0.52–0.87) on Day 2. |
| 2 | Zhenli Li 2024 | Model 2 | C statistic：0.807 (95% CI: 0.707−0.908). | Based on bootstrap: C statistic：0.779 (95% CI: 0.677−0.865).  Calibration curve for 40-day major adverse cardiovascular events showed good degree of fitting。 DCA indicated that if the threshold probability of a patient or doctor was > 5% and < 60%, using this nomogram to predict the 40-day major adverse cardiovascular events added more benefits. | None |
| 3 | Rui Lu 2024 | Model 1 | AUC-ROC：0.967 (95%CI：0.916~1.000); Youden Index：0.882; sensitivity：88.2%; specificity：100%. | None | None |
|  |  | Model 2 | None | Based on leave-one-out cross validation：  AUC-ROC: 0.846; accuracy: 0.771; sensitivity: 0.706; specificity: 0.833.  Calibration curve showed good agreement between predicted and observed outcomes. | None |

Table 4-5 Performance of prediction model for the risk of major adverse cardiovascular/cardiac events following immune checkpoint inhibitor-related cardiovascular toxicity (continued)

| **No** | **Author (year)** | **Model number** | **Apparent performance** | **Internal validation performance** | **External validation performance** |
| --- | --- | --- | --- | --- | --- |
| 3 | Rui Lu 2024 | Model 3 | None | Based on leave-one-out cross validation：  AUC-ROC: 0.892; accuracy: 0.829; sensitivity: 0.706; specificity:0.994.  Calibration curve showed good agreement between predicted and observed outcomes. | None |
|  |  | Model 4 | None | Based on leave-one-out cross validation：  AUC-ROC 0.827; accuracy: 0.714; sensitivity: 0.706; specificity: 0.722.  Calibration curve showed the calibration performance was poorer than that of Model 2 and Model 3. | None |
|  |  | Model 5 | None | Based on leave-one-out cross validation：  AUC-ROC 0.784; accuracy: 0.829; sensitivity: 0.824; specificity: 0.833.  Calibration curve showed the calibration performance was poorer than that of Model 2 and Model 3. | None |
| CI:confidence interval; DCA: decision curve analysis; AUC-ROC: area under receiver operating curves. | | | | | |

Table 4-6 Performance of diagnostic prediction model for grading the severity of immune checkpoint inhibitor-related cardiovascular toxicity

| **No** | **Author (year)** | **Model number** | **Apparent performance** | **Internal validation performance** | **External validation performance** |
| --- | --- | --- | --- | --- | --- |
| 1 | Zhuoling Zheng 2026 | Model 2 | AUC-ROC：0.769 (95% CI: 0.655-0.882). | Based on bootstrap: AUC-ROC: 95% CI: 0.654-0.861. | None |
| 2 | Zhenli Li 2024 | Model 1 | C statistic：0.757 (95% CI: 0.646− 0.869). | Based on bootstrap: C statistic：0.752 (95% CI: 0.635−0.866). Calibration curve for severe ICI‑related myocarditis was very close to the actual result. DCA indicated that if the threshold probability of a patient or doctor was > 20% and < 85%, using this nomogram to predict the severity of ICI‑related myocarditis might add more benefits. | None |
| AUC-ROC: area under receiver operating curves; CI:confidence interval; DCA: decision curve analysis. | | | | | |

Figure 1-1 Quality, risks of bias and applicability assessment of prediction model for the occurrence risk of overall immune checkpoint inhibitor-related cardiovascular toxicity

Figure 1-1 (1) Quality and applicability assessment of prediction model for the occurrence risk of overall immune checkpoint inhibitor-related cardiovascular toxicity

Figure 1-1 (2) Risks of bias and applicability assessment of prediction model for the occurrence risk of overall immune checkpoint inhibitor-related cardiovascular toxicity

Figure 1-2 Quality, risks of bias and applicability assessment of prediction model for the occurrence risk of immune checkpoint inhibitor-related myocarditis

Figure 1-2 (1) Quality and applicability assessment of prediction model for the occurrence risk of immune checkpoint inhibitor-related myocarditis

Figure 1-2 (2) Risks of bias and applicability assessment of prediction model for the occurrence risk of immune checkpoint inhibitor-related myocarditis

Figure 1-3 Quality, risks of bias and applicability assessment of prediction model for the occurrence risk of immune checkpoint inhibitor-related venous thromboembolism

Figure 1-3 (1) Quality and applicability assessment of prediction model for the occurrence risk of immune checkpoint inhibitor-related venous thromboembolism

Figure 1-3 (2) Risks of bias and applicability assessment of prediction model for the occurrence risk of immune checkpoint inhibitor-related venous thromboembolism

Figure 1-4 Quality, risks of bias and applicability assessment of prediction model for the risk of mortality following immune checkpoint inhibitor-related cardiovascular toxicity

Figure 1-4 (1) Quality and applicability assessment of prediction model for the risk of mortality following immune checkpoint inhibitor-related cardiovascular toxicity

Figure 1-4 (2) Risks of bias and applicability assessment of prediction model for the risk of mortality following immune checkpoint inhibitor-related cardiovascular toxicity

Figure 1-5 Quality, risks of bias and applicability assessment of prediction model for the risk of major adverse cardiovascular/cardiac events following immune checkpoint inhibitor-related cardiovascular toxicity

Figure 1-5 (1) Quality and applicability assessment of prediction model for the risk of major adverse cardiovascular/cardiac events following immune checkpoint inhibitor-related cardiovascular toxicity

Figure 1-5 (2) Risks of bias and applicability assessment of prediction model for the risk of major adverse cardiovascular/cardiac events following immune checkpoint inhibitor-related cardiovascular toxicity

Figure 1-6 Quality, risks of bias and applicability assessment of diagnostic prediction model for grading the severity of immune checkpoint inhibitor-related cardiovascular toxicity

Figure 1-6 (1) Quality and applicability assessment of diagnostic prediction model for grading the severity of immune checkpoint inhibitor-related cardiovascular toxicity

Figure 1-6 (2) Risks of bias and applicability assessment of diagnostic prediction model for grading the severity of immune checkpoint inhibitor-related cardiovascular toxicity
